# Novel epigenetic loci identified from an epigenome-wide association study underlying brain structural changes in bipolar disorder

**DOI:** 10.1017/S003329172610347X

**Published:** 2026-03-05

**Authors:** Hyun-Ho Yang, Kyu-Man Han, Youbin Kang, Daun Shin, Woo-Suk Tae, Mi-Ryung Han, Byung-Joo Ham

**Affiliations:** 1 Division of Life Sciences, College of Life Sciences and Bioengineering, Incheon National University, Incheon, Republic of Korea; 2 Department of Psychiatry, Korea University Anam Hospital, Korea University College of Medicine, Seoul, Republic of Korea; 3 Brain Convergence Research Center, Korea University College of Medicine, Seoul, Republic of Korea; 4 Department of Biomedical Sciences, Korea University College of Medicine, Seoul, Republic of Korea; 5 Institute for New Drug Development, College of Life Science and Bioengineering, Incheon National University, Incheon, Republic of Korea

**Keywords:** bipolar disorder, cortical thickness, DNA methylation, Epigenome-wide association study, Neuroimaging-epigenetic study

## Abstract

**Background:**

DNA methylation influences gene–environment interactions and brain development in bipolar disorder (BD). We aimed to identify BD-associated epigenetic loci and examine their associations with brain structural variation.

**Methods:**

We conducted an epigenome-wide association study (BD group, n = 90; healthy controls group, n = 161) to identify BD-associated DNA methylation loci, and we additionally performed copy number alteration and functional enrichment analyses. The correlations between epigenetic loci and cortical thickness (CT) were assessed using Pearson’s partial correlation analysis, and the co-methylation effect of the epigenetic loci identified in the neuroimaging–epigenetic analysis was investigated.

**Findings:**

A total of 156 differentially methylated positions (DMPs) and 7 differentially methylated regions were identified, and the genes associated with them were observed to be enriched in biological processes related to muscle hypertrophy and neuronal activity. Significant correlations between the methylation levels of 13 DMPs associated with three genes (*miR886*, *PLEC1*, and *ICAM5*) and the CT of the right postcentral gyrus and inferior frontal gyrus were identified. Specifically, 10 DMPs associated with the CpG island in the upstream region of the *miR886* gene showed negative correlations with the right postcentral gyrus CT, implicating *miR886*-associated CpG-island methylation in regional cortical thinning.

**Conclusion:**

Epigenetic changes might play an important role in brain structural changes in BD. These multimodal findings nominate *miR886*-related methylation as a candidate molecular correlate of cortical thinning and warrant replication and mechanistic follow-up in larger, state-diverse cohorts.

## Introduction

Bipolar disorder (BD), a complex disorder characterized by severe fluctuations in mood states, recurrent episodes of mania and depression, and a high risk of suicide, can have a strong negative impact on social activities, cognition, and overall quality of life (McIntyre et al., 2020). The overall prevalence of BD is approximately 1%, with a heritability rate of approximately 60% (Bessonova et al., 2020; Merikangas et al., 2011; Smoller & Finn, 2003; Song et al., 2018). However, in a large-scale genome-wide association study conducted by the Psychiatric Genomics Consortium, genetic variations accounted for approximately 19% of the phenotypic variance in BD (Mullins et al., 2021). Additionally, a substantial body of research indicates that BD is influenced by various genetic and environmental factors (e.g. preterm birth, adverse childhood experiences, substance abuse) (Anand et al., 2015; Marangoni, Hernandez, & Faedda, 2016; Musci, Augustinavicius, & Volk, 2019; Nosarti et al., 2012; Rodriguez et al., 2021). Accordingly, DNA methylation, which is influenced by several environmental factors and dynamically regulates gene expression, may be relevant for understanding the pathophysiology of BD (Uher, 2014).

DNA methylation is a mechanism of epigenetic regulation regulated by DNA methyltransferase and involves the addition of methyl groups to the C5 position of cytosine (Mattei, Bailly, & Meissner, 2022). Epigenetic patterns in the nervous system can be influenced by various environmental risk factors, and abnormal epigenetic patterns can affect gene expression (Moore, Le, & Fan, 2013). It is possible that similar changes reflect gene-environment interactions associated with BD etiology and affect the expression patterns of relevant genes, which could then be potential candidates for regulating the neurobiological mechanisms involved in the pathogenesis of BD (Fries et al., 2016). Epigenome-wide association studies (EWASs) have emerged as a successful methodology for identifying changes in epigenetic patterns associated with various diseases. Nevertheless, despite the growing body of evidence that suggests associations between the instability of DNA methylation patterns and the development of BD, evidence regarding epigenetic variations associated with the development of BD remains limited compared to that for other psychiatric disorders (Bundo et al., 2021; Fries et al., 2016; Hesam-Shariati et al., 2022; Legrand, Iftimovici, Khayachi, & Chaumette, 2021).

Considering that DNA methylation plays an essential role in brain maturation and function, structural and functional changes in brain networks may mediate the association between epigenetic variations and BD development (Gapp, Woldemichael, Bohacek, & Mansuy, 2014). The cortical thickness (CT), the highly folded neuron sheets that form the outer layer of the brain, is considered a direct quantitative indicator of cerebral cortex integrity and morphology (Fischl & Dale, 2000; Rebsamen et al., 2020). It reflects geographically preserved information and is associated with various important parameters, such as neuronal and glial cell numbers, dendritic arborization, neuron size, and extracellular space size (Hanford, Nazarov, Hall, & Sassi, 2016; Narr et al., 2007). CT is particularly relevant to the pathophysiology of BD and is one of the most intensively researched neuroimaging parameters in relation to genetic factors in BD (Harrison, Colbourne, & Harrison, 2020). For instance, a large-scale longitudinal study by the Enhancing Neuro Imaging Genetics through Meta-Analysis (ENIGMA) BD working group revealed slower thinning of fusiform and hippocampal CT in patients with BD, and a negative correlation between the frequency of (hypo)manic episodes and prefrontal CT (Abé et al., 2022). Additionally, a recent meta-analysis revealed significant cortical thinning across broad areas of the brain in patients with BD, including the bilateral anterior cingulate cortex, left Rolandic operculum, left transverse temporal gyrus, left inferior frontal gyrus, and left superior frontal gyrus (Zhu et al., 2022).

Several studies have investigated the associations between brain structure and genetic variations in patients with BD, including one study on the associations between changes in brain structure and the methylation level of the *OXTR* gene, which is linked to social cognition abilities and emotion processing (Abé et al., 2020; Han et al., 2019; Jiang et al., 2023; Kennedy et al., 2022; Rubin et al., 2016). However, to the best of our knowledge, the EWAS approach has not yet been used to examine the changes in brain structure in BD. This study aimed to identify the differences in epigenetic patterns associated with the pathophysiology of BD and investigate their correlation with changes in CT. First, we investigated significant differences in epigenetic patterns using EWAS at the whole-genome level. Second, we explored structural changes in CT using T1-weighted magnetic resonance imaging (MRI) data. Thereafter, correlations between CT and the methylation levels of epigenetic loci identified based on the EWAS were examined. Finally, we used functional enrichment analysis to identify significantly enriched pathways and functions using the gene list obtained from the EWAS and performed copy number alteration (CNA) analysis to detect structural variations associated with the pathophysiology of BD.

## Materials and methods

### Study participants

This study included 161 healthy controls (HCs) and 90 patients with BD as confirmed by at least two experienced board-certified psychiatrists (Ham B.J. and Han K.M.) based on a standardized clinical interview (the Structured Clinical Interview for the 5th edition of the Diagnostic and Statistical Manual of Mental Disorders Axis I disorders) (First, Williams, Karg, & Spitzer, 2016). Patients with BD were recruited between January 2015 and August 2021 at the outpatient psychiatric clinic of Korea University Anam Hospital in Seoul, Republic of Korea. Self-reported information was used to confirm that the ancestry of each participant for the last three generations was Korean. Additionally, no samples were identified as genetic outliers through principal component analysis and Mahalanobis distance (Supplementary Method 1). The inclusion criteria for the BD group were as follows: (i) a diagnosis of bipolar I or II disorder according to DSM-5; (ii) age between 19 and 69 years; and (iii) current euthymic or depressive state, operationally defined as a score of ≤12 on the 11-item Young Mania Rating Scale (YMRS), indicating the absence of (hypo)manic symptoms (Macellaro et al., 2025; Suppes et al., 2005). Patients meeting criteria for (hypo)mania were explicitly excluded. This restriction was applied to reduce mood-state–related heterogeneity (Abé et al., 2015) and to ensure structural MRI data quality, as in-scanner head motion can systematically bias cortical morphometric estimates (Reuter et al., 2015). The exclusion criteria were: (i) comorbid diagnosis of major psychiatric disorders (including personality and substance use disorders); (ii) high suicidal risk requiring immediate inpatient treatment; (iii) history of a serious or unstable medical illness; (iv) primary neurological illness (e.g. Parkinson’s disease, cerebrovascular disease, or epilepsy); (v) pregnancy or nursing; and (vi) any contraindications for MRI (Han et al., 2019). HCs were recruited via advertisements during the same period. In addition to the aforementioned criteria, the absence of a history of psychiatric disorders was used as an additional criterion for their recruitment.

The severity of depressive symptoms was assessed using the 17-item Hamilton Depression Rating Scale (HDRS) (Hamilton, 1960). The 11-item YMRS was used to assess the manic symptoms of patients with BD (Young, Biggs, Ziegler, & Meyer, 1978). All patients were confirmed as being right-handed using the Edinburgh Handedness Test (Oldfield, 1971). For the BD group, the duration of illness was defined as the lifetime cumulative number of months of depressive and (hypo)manic episode(s) using the life-chart methodology.

This study was approved by the Institutional Review Board of Korea University Anam Hospital (*2017AN0185*). All participants provided written informed consent before being included in the study.

### Data acquisition and processing

A combination of two datasets was used: (i) genomic data for biomarker identification and (ii) brain MRI data. To measure the DNA methylation levels of peripheral blood of each participant, the Infinium MethylationEPIC BeadChip (Illumina Inc.; San Diego, CA, USA) was used according to the manufacturer’s protocol (Supplementary Method 2). After removing low-quality samples and probes, the beta-mixture quantile normalization and ComBat algorithms were applied to eliminate technical biases (Supplementary Method 3) (Johnson, Li, & Rabinovic, 2007). The composition of white blood cell was estimated using the FlowSorted.Blood.EPIC R package to control potential bias from cell type heterogeneity in DNA methylation (Supplementary Method 4) (Houseman et al., 2012; Salas et al., 2018). The beta (β) value – range from 0 to 1 – was used to represent the methylation level.

Among 251 participants, 82 patients with BD and 154 HCs underwent brain MRI to acquire T1-weighted images (Supplementary Method 5). FreeSurfer version 7.2 (Laboratory for Computational Neuroimaging, Athinoula A. Martinos Center for Biomedical Imaging, Charlestown, MA, USA; http://surfer.nmr.mgh.harvard.edu) was used to measure the thickness of 38 cortical gyri in each hemisphere according to the atlas by Destrieux et al. (Supplementary Method 6) (Destrieux, Fischl, Dale, & Halgren, 2010). CT was defined as the minimum distance from the boundary of gray/white matter to the pial surface.

### Differential methylation analysis

Differential methylation analysis for comparing HCs and patients with BD was conducted at both the probe and region levels. Age and sex were considered as covariates. Differentially methylated probes (DMPs) were identified using the limma R package (Ritchie et al., 2015). Type I errors were controlled using false discovery rate (FDR) correction. Significant DMPs were defined as those with a FDR ≤ 0.05 and an absolute Δβ value ≥0.07 based on statistical power estimation using the pwrEWAS R package (Supplementary Method 7) (Supplementary Figure S1) (Graw, Henn, Thompson, & Koestler, 2019). For region-level analysis, the DMRcate R package was used to identify differentially methylated regions (DMRs) based on previously recommended parameters (lambda = 500; C = 5) (Mallik et al., 2019; Peters et al., 2015). Significant DMRs were identified based on the cutoff of Stouffer P-value ≤0.05, absolute mean Δβ value ≥0.05, and the number of probes in region ≥7. The GRCh38/hg38 reference genome was used to represent the position of each DMP and DMR.

### Neuroimaging–epigenetic analysis

First, one-way analysis of covariance was used to explore the differences in CTs in 76 cortical gyri of the bilateral hemispheres between patients with BD and HCs. Age, sex, total intracranial cavity volume (TICV), and years of education were controlled as covariates. Second, we performed correlation analyses between significant DMPs from the EWAS and CTs for the 76 cortical gyri to investigate brain structural correlates of epigenetic signatures in the BD and HC groups. Pearson’s partial correlation analysis was conducted to explore significant relationships between CTs and the DNA methylation levels of DMPs (Han et al., 2022). In the BD group, age, sex, years of education, TICV, HDRS score, YMRS score, and illness duration were considered as covariates; age, sex, years of education, and TICV were considered as covariates in the HC group. The Benjamini–Hochberg approach was equally applied for both analyses (FDR ≤ 0.05).

### Copy number analysis in patients with BD

To identify CNAs in BD, the circular binary segmentation algorithm in the ChAMP R package was used, with HCs as the reference (Olshen, Venkatraman, Lucito, & Wigler, 2004; Tian et al., 2017). Frequently recurring focal alterations were identified using the Genomic Identification of Significant Targets in Cancer (GISTIC) 2.0 algorithm (Mermel et al., 2011). Significant focal amplifications and deletions were defined using the |copy number| ≥ 0.5 and FDR ≤ 1.0×10^−3^ cutoffs. Genomic positions of all focal alterations are represented based on the GRCh38/hg38 reference genome.

### Gene ontology and pathway enrichment analysis

To gain insights into the relevant biological functions, Gene ontology (GO) and Kyoto Encyclopedia of Genes and Genomes (KEGG) pathway enrichment analyses were performed using the GOmeth algorithm (Maksimovic, Oshlack, & Phipson, 2021; Phipson, Maksimovic, & Oshlack, 2016). Both functional enrichment analyses were conducted for significant DMPs (FDR ≤ 0.05 and |Δβ| ≥ 0.07). Significantly enriched GO terms and KEGG pathways were identified using the unadjusted P-value ≤ 0.05 and minimum number of genes associated with DMPs ≥ 2 cutoffs.

### Co-methylation analysis

Given that the primary neuroimaging–epigenetic analyses identified significant CT–methylation associations in the right postcentral gyrus and right pars triangularis, we conducted weighted gene co-methylation network analysis (WGCNA) as an exploratory follow-up to further characterize coordinated methylation patterns and to identify potential hub CpG sites related to regional CT. Specifically, to expand upon the findings of neuroimaging–epigenetic analysis and to further investigate potential CpG sites, we explored key co-methylation modules that have significant correlations with the CTs of the right postcentral gyri and pars triangularis using the WGCNA R package (P-value ≤ 2.78×10^−3^) (Supplementary method 8) (Langfelder & Horvath, 2008). Hub CpG sites were identified based on the criteria of | module membership | ≥0.9 and | gene significance | ≥0.3 for the key module. Functional enrichment analysis for the CpG sites in the key modules was conducted using the same method as described above. R version 4.2.3 was used for all statistical analyses.

## Results

### Differential methylation analysis results

The sociodemographic and clinical characteristics of participants (90 patients with BD and 161 HCs) who satisfied the study criteria are listed in Table 1. Age, HDRS scores, and years of education differed significantly between the two groups. The methylation levels of 729,166 probes, excluding those that did not meet the inclusion criteria, were included in the downstream analysis.

**Table 1.** Demographic and clinical characteristics of patients with BD and HCs

Characteristics	BD (n = 90)	HC (n = 161)	P-value (t, χ^2^)
Age (mean ± SD)	32.86 ± 10.63	38.41 ± 13.94	<0.001^a^ (t = –3.539)
Sex (female/male)	58/32	104/57	1^b^ (χ2 = 0)
Education years (mean ± SD)	13.81 ± 2.49	15.04 ± 2.26	<0.001^a^ (t = –3.859)
HDRS–17 score (mean ± SD)	8.11 ± 5.64	0.99 ± 1.74	<0.001^a^ (t = 11.662)
YMRS score (mean ± SD)	1.18 ± 2.08	NA	NA
Remission state/depressive state	47/43	NA	NA
Illness duration (mean ± SD) (months)	27.01 ± 20.68	NA	NA
Drug-treated patients (n)	90	NA	NA
**Medication (n)**			
**Antidepressants**			
None	53		
SSRI	16	NA	NA
SNRI	6		
NDRI	10		
NaSSA	2		
Etc	2		
Combination of AD	1		
**Mood stabilizer**			
None	10		
Li	17		
AED	57		
Combination of Li and AED	3		
Combination of AEDs	3		
**Antipsychotics**			
None	14		
AP	47		
Combination of AP	29		

*Note:* BD, bipolar disorder; HC, healthy control; SD, standard deviation; HDRS-17, 17-item Hamilton Depression Rating Scale; YMRS, Young Mania Rating Scale; SSRI, selective serotonin reuptake inhibitor; SNRI, serotonin and norepinephrine reuptake inhibitor; NDRI, norepinephrine-dopamine reuptake inhibitor; NaSSA, noradrenergic and specific serotonergic antidepressant; Combination of AD, combinations of two or more types of antidepressant; APs, antipsychotics; ADs, antidepressants; Li, lithium; AED, antiepileptic mood stabilizer; Combination of AEDs, combinations of two or more types of antiepileptic mood stabilizer; Combination of APs, combinations of two or more types of antipsychotics.

a P-values for comparisons of age, education years, and HDRS scores were obtained using an independent *t*-test.

b P-values for the distribution of sex were obtained using a chi-squared test.

**Figure 1.**
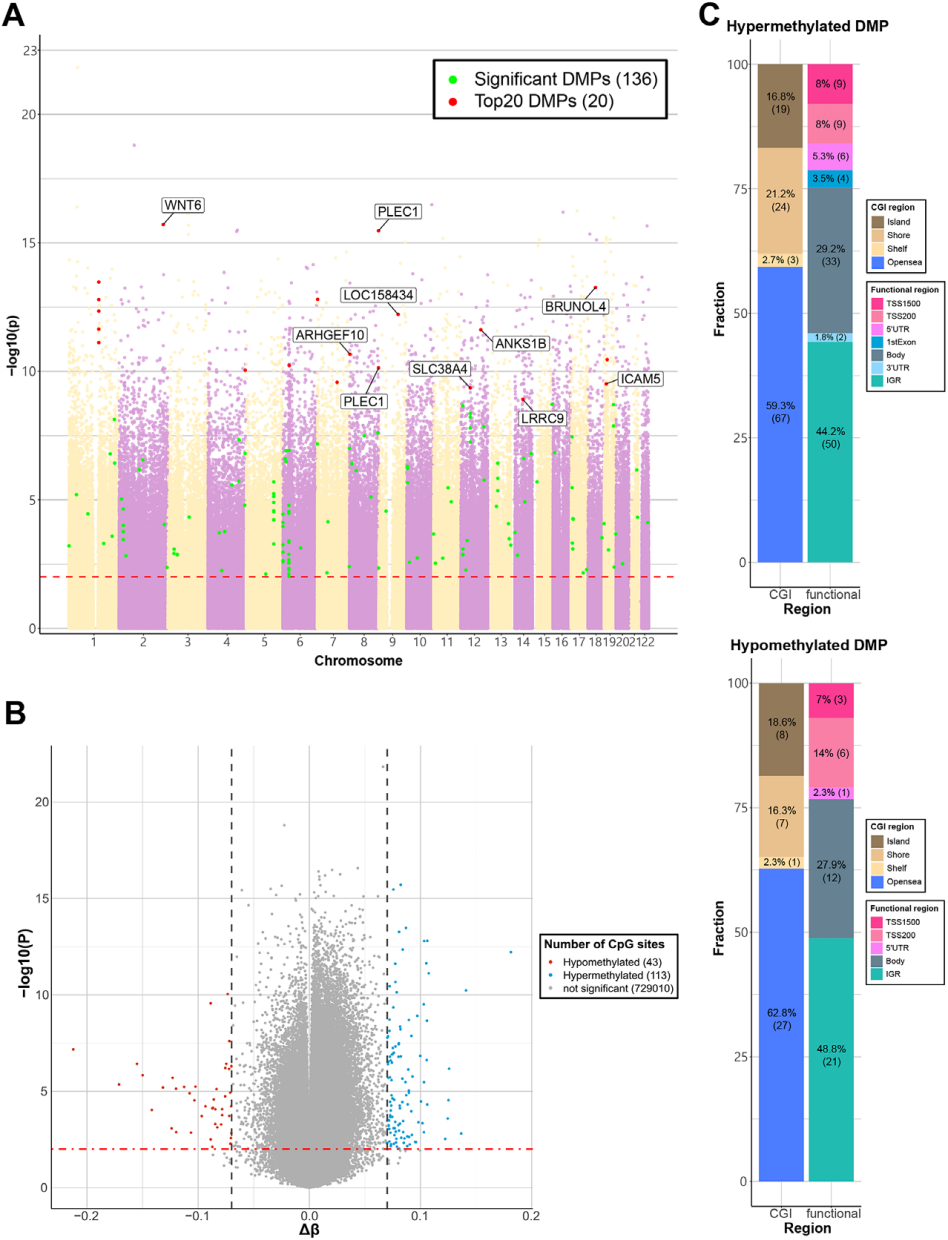
Visualization of EWAS results for the BD and HC groups. (a) Manhattan plot of EWAS results. The x-axis shows chromosomes using two different colors, while the y-axis shows −log10(P-value). The horizontal dashed red line indicates the Benjamini–Hochberg corrected P-value of 0.05 (FDR ≤ 0.05). The top 20 significant DMPs are represented as red dots, and the gene names associated with each CpG site are labeled. The remaining DMPs are represented as green dots. (b) Volcano plot of EWAS result. The Δβ and −log10(P-value) are shown on the x-axis and y-axis, respectively. The horizontal dot-dashed line indicates the Benjamini–Hochberg corrected P-value of 0.05 (FDR≤0.05). The vertical dashed dark gray lines indicate an absolute Δβ value of 0.07. Blue dots represent hypermethylated DMPs, and red dots represent hypomethylated DMPs, while gray dots represent non-significant probes. (c) Stacked box plot of 156 DMPs (FDR ≤ 0.05, |Δβ| ≥ 0.07). The percentage of functional and CGI regions for hypermethylated (top) and hypomethylated (bottom) DMPs are illustrated.

A total of 156 CpG sites were identified as significant DMPs in the EWAS comparing the BD and HC groups (Supplementary Table S1) (FDR ≤ 0.05, |Δβ| ≥ 0.07). The top-ranked DMPs included cg13903421 (*WNT6*), cg04255391 (*PLEC1*), cg13392957 (intergenic region), and cg03356492 (*BRUNOL4*). The top 20 DMPs are listed in Table 2 and highlighted in Figure 1a. Of the 156 DMPs, 113 were hypermethylated, and 43 were hypomethylated (Figure 1b). The fractions of genomic regions with hypermethylated and hypomethylated DMPs are shown in Figure 1c. Epigenetic variation at CpG sites located in gene bodies and promoters can affect transcription (Wang et al., 2022). The high proportion of DMPs in gene promoters and gene bodies (55.8% of hypermethylated and 51.2% of hypomethylated DMPs) suggests that the transcription of genes associated with these DMPs is altered in patients with BD (Figure 1c).

**Table 2.** Top 20 DMPs obtained from EWAS

CpG site	P-value	FDR^a^	Δβ	CHR	Position^b^	Gene	Functional region^c^	CGI region^d^
cg13903421	1.94E-16	1.34E-11	0.082	chr2	218873992	*WNT6*	3’UTR	Island
cg04255391	3.41E-16	1.78E-11	0.076	chr8	143934229	*PLEC1*	Body	Shore
cg13392957	3.34E-14	3.93E-10	0.087	chr1	148310171		IGR	Island
cg03356492	5.44E-14	5.30E-10	0.081	chr18	37482575	*BRUNOL4*	Body	Shelf
cg01938825	1.57E-13	9.56E-10	0.106	chr7	1524072		IGR	OpenSea
cg03242265	1.61E-13	9.70E-10	0.103	chr1	148310364		IGR	Island
cg13067553	4.51E-13	1.99E-09	0.084	chr1	148310344		IGR	Island
cg03249723	6.10E-13	2.49E-09	0.181	chr9	96117775	*LOC158434*	TSS1500	OpenSea
cg23206461	2.29E-12	6.44E-09	0.105	chr1	148310008		IGR	Island
cg06312072	2.43E-12	6.69E-09	0.077	chr12	98893351	*ANKS1B*	Body	Shore
cg18140268	7.56E-12	1.53E-08	0.107	chr1	148309719		IGR	Shore
cg05833946	2.15E-11	3.21E-08	0.077	chr8	1899833	*ARHGEF10*	Body	OpenSea
cg21068178	3.51E-11	4.47E-08	0.072	chr19	15130012		IGR	OpenSea
cg00020172	5.92E-11	6.43E-08	0.141	chr6	31082596		IGR	OpenSea
cg02331830	7.35E-11	7.61E-08	0.079	chr8	143934120	*PLEC1*	Body	Shore
cg17984267	9.07E-11	8.79E-08	−0.073	chr4	185007740		IGR	Shelf
cg07283849	2.68E-10	1.91E-07	−0.089	chr7	97771750		IGR	OpenSea
cg10604476	3.09E-10	2.12E-07	0.103	chr19	10293232	*ICAM5*	Body	Island
cg21428710	4.47E-10	2.75E-07	0.075	chr12	46826014	*SLC38A4*	TSS200	OpenSea
cg16597280	1.24E-09	5.69E-07	0.097	chr14	59965957	*LRRC9*	Body	OpenSea

*Note:* DMP, differentially methylated probe; BD, bipolar disorder; HC, healthy control; Δβ, the average β value of patients with BD minus the average β value of HCs; CGI, CpG island; CHR, chromosome.

a The Benjamini–Hochberg (BH) approach was used (FDR≤0.05).

b UCSC GRCh38/hg38.

c CpGs located in functional genomic regions, TSS1500, 200–1500 bases upstream of the transcriptional start site; TSS200, 0–200 bases upstream of the transcriptional start site; Body, region between the ATG and stop codons; 3′UTR, region between the stop codon and the poly A signal; IGR, intergenic region.

d CpGs located in CpG islands, Shelf, 2–4 kb from a CpG island; Shore, 0–2 kb from a CpG island; OpenSea, >4 kb from a CpG island; Island, CpG island.

Differential methylation analysis at the region level identified 7 DMRs considering sex and age as covariates (Supplementary Table S2). The most significant DMR consisted of 12 CpG sites and was located between 1500 bases upstream of the transcriptional start site (TSS1500) and the first exon of the *SLC38A4* gene (Stouffer P-value = 6.54 × 10^−51^; mean Δβ = 0.054).

### Functional enrichment analysis

Functional enrichment analysis of the 156 DMPs identified by the EWAS showed that the genes associated with them are enriched in 86 GO terms, comprising 62 biological processes, 17 cellular components, seven molecular functions, and one pathway (Supplementary Table S3). However, after controlling for multiple testing errors, no ontology terms or pathways remained significant (FDR ≤ 0.05).

### Identification of significant CNAs

Fifteen focal amplifications and fourteen focal deletions were identified using the GISTIC algorithm to detect frequently recurring focal CNAs (Supplementary Figure S2). The recurrent focal amplifications identified in patients with BD included human leukocyte antigen (HLA)-related regions (6p21.33, 6p22.1, 6p21.32), 8p23.1, and 1p13.3. With regard to focal deletions, 8p23.1, 1q31.3, and 16p12.2 were identified as the most frequently recurring regions (Supplementary Table S4).

### CT alterations in BD

Brain MRI data from 82 of the 90 patients with BD and 154 of the 161 HCs were used to identify changes in CT in 76 brain regions (Supplementary Table S5). After adjusting for age, sex, TICV, and years of education, consistent CT thinning was observed in 49 cortical regions in patients with BD compared to that in HCs (FDR ≤ 0.05). Brain regions associated with the prefrontal cortex, such as the frontomarginal, transverse frontopolar, and straight gyri, were identified as those with the most significant thinning. Regions in the somatosensory cortex (e.g. the postcentral gyrus) showed a significant decrease in CT (Supplementary Table S6). None of the cortical regions showed significant thickening in the BD group compared to that in the HC group (Supplementary Table S6).

### Neuroimaging–epigenetic analysis

**Figure 2.**
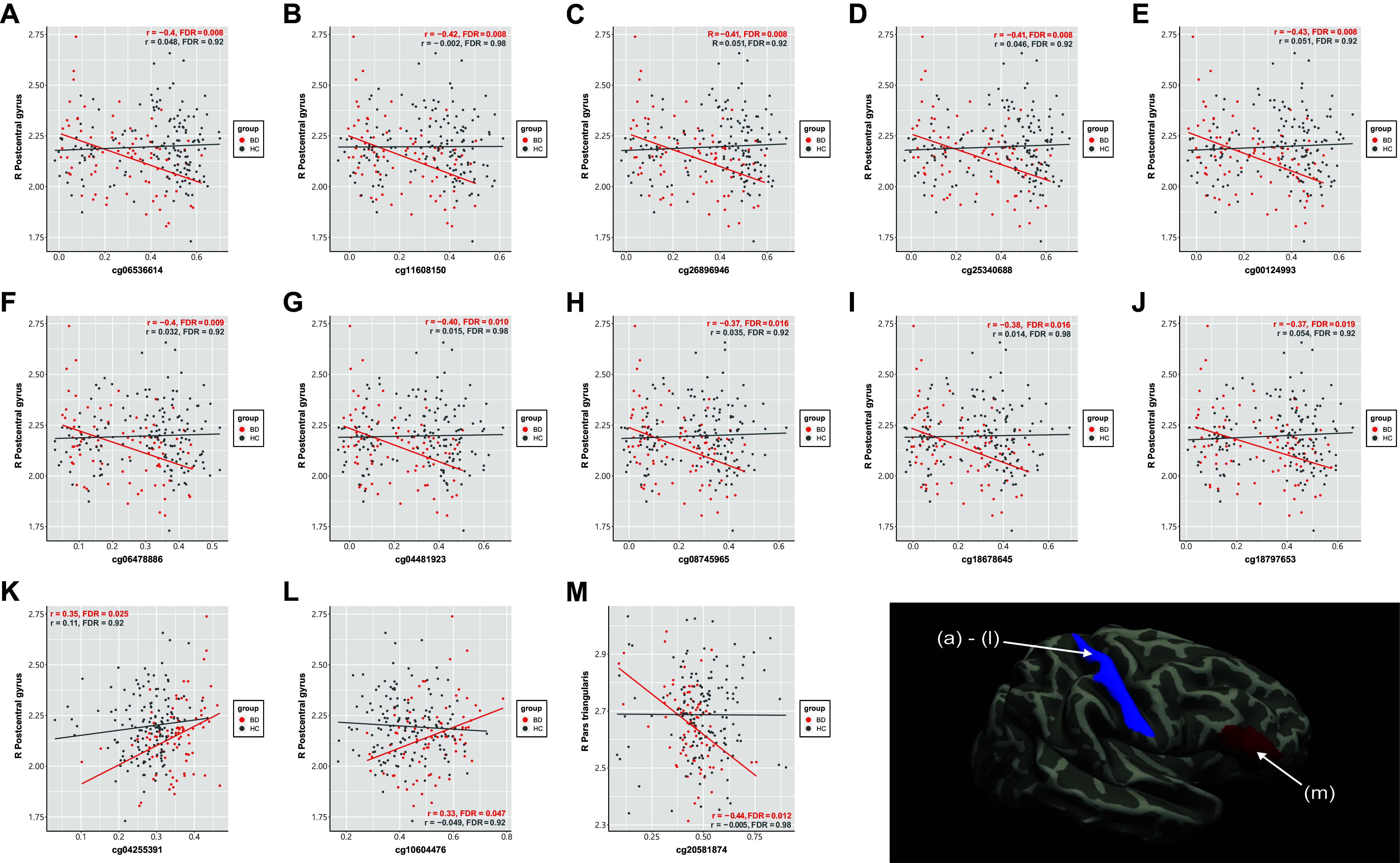
Scatterplots and a brain image of neuroimaging–epigenetic analysis. Each scatterplot represents a significant correlation between the methylation level of CpG sites ((a) cg06536614, (b) cg11608150, (c) cg26896946, (d) cg25340688, (e) cg00124993, (f) cg06478886, (g) cg04481923, (h) cg08745965, (i) cg18678645, (j) cg18797653, (k) cg04255391, (l) cg10604476, and (m) cg20581874) and the cortical thickness of brain regions in patients with BD. The x-axis shows the methylation level of a CpG site, while the y-axis shows the cortical thickness of a brain region. Dots represent patients with BD (red) and HCs (gray), and lines represent the correlation between the methylation level of CpG sites and the cortical thickness of brain regions. The brain image represents cortical regions, based on the Destrieux atlas, that showed a significant correlation with the methylation levels of 13 DMPs in the neuroimaging–epigenetic analysis. The scatterplots of the corresponding cortical regions are denoted by letters ((a)–(l) right postcentral gyrus, (m) right pars triangularis).

Pearson’s partial correlation analysis was used to assess the relationships between brain structural variations and epigenetic variations, specifically the CTs of 76 brain regions and methylation levels of 156 DMPs associated with BD. We identified correlations between the CT of the right postcentral gyrus and the methylation levels of 12 DMPs (cg06536614, cg11608150, cg26896946, cg25340688, cg00124993, cg06478886, cg04481923, cg08745965, cg18678645, cg18797653, cg04255391, and cg10604476) and between the CT of the right pars triangularis (inferior frontal gyrus) and the methylation level of one DMP (cg20581874) (Table 3). None of these relationships were statistically significant in the HC group (Figure 2). According to the UCSC CpG island annotations, 10 of the 12 DMPs that correlated with the right postcentral gyrus CT are located near a CpG island in the upstream region of the *miR886* gene (chr5:136,080,515–136,080,786).

**Table 3.** Neuroimaging–epigenetic analysis in patients with BD

Cortical regions	CpG site (functional region^a^; Δβ)	CHR	Position^b^	Gene	r	P-value	FDR^c^
R Postcentral gyrus	cg06536614 (TSS200; −0.123)	chr5	136080692	*miR886*	−0.409	2.69E-04	8.38E-03
R Postcentral gyrus	cg11608150 (IGR; −0.102)	chr5	136080259		−0.415	2.13E-04	8.38E-03
R Postcentral gyrus	cg26896946 (TSS200; −0.113)	chr5	136080716	*miR886*	−0.413	2.29E-04	8.38E-03
R Postcentral gyrus	cg25340688 (TSS200; −0.120)	chr5	136080709	*miR886*	−0.411	2.53E-04	8.38E-03
R Postcentral gyrus	cg00124993 (TSS200; −0.108)	chr5	136080723	*miR886*	−0.429	1.21E-04	8.38E-03
R Postcentral gyrus	cg06478886 (IGR; −0.070)	chr5	136080340		−0.403	3.35E-04	8.71E-03
R Postcentral gyrus	cg04481923 (Body; −0.103)	chr5	136080516	*miR886*	−0.396	4.42E-04	9.84E-03
R Postcentral gyrus	cg08745965 (TSS1500; −0.086)	chr5	136080840	*miR886*	−0.375	9.31E-04	1.61E-02
R Postcentral gyrus	cg18678645 (TSS200; −0.093)	chr5	136080642	*miR886*	−0.376	8.95E-04	1.61E-02
R Postcentral gyrus	cg18797653 (TSS1500; −0.079)	chr5	136080924	*miR886*	−0.367	1.20E-03	1.87E-02
R Postcentral gyrus	cg04255391 (Body; 0.076)	chr8	143934229	*PLEC1*	0.356	1.74E-03	2.46E-02
R Postcentral gyrus	cg10604476 (Body; 0.103)	chr19	10293232	*ICAM5*	0.332	3.61E-03	4.69E-02
R Pars triangularis	cg20581874 (IGR; −0.072)	chr14	20723595		−0.439	8.01E-05	1.25E-02

*Note:* BD, bipolar disorder; HC, healthy control; Δβ, the average β value of patients with BD minus the average beta value of HCs; FDR, false discovery rate; R, right hemisphere; CHR, chromosome.

a CpGs locate in functional genomic regions, TSS1500, 200–1500 bases upstream of the transcriptional start site; TSS200, 0–200 bases upstream of the transcriptional start site; Body, between the ATG and stop codon; IGR, intergenic region.

b UCSC GRCh38/hg38.

c the Benjamini–Hochberg (BH) approach was used (FDR ≤ 0.05).

### Co-methylation analysis

Finally, we examined the co-methylation effects of the 13 DMPs identified using the neuroimaging–epigenetic analysis. A total of 11,677 CpG sites were included to estimate co-methylation effects (FDR ≤ 0.05 and |Δβ| ≥ 0.02). Eighteen modules were identified after merging similar modules (Supplementary Figure S3a). Of these, the MEs of four modules were observed to be correlated with the CTs of the right postcentral gyrus and right pars triangularis (P-value ≤ 2.78 × 10^−3^) (Supplementary Figure S3b). Of the 13 DMPs, 10 DMPs associated with the CpG island in the upstream region of the *miR886* gene were included in the 12 hub CpG sites of the lightgreen module (Supplementary Figure S3c; Supplementary Table S7). Additionally, the lightgreen module did not show significant correlations with any of the following variables: two batch effects (slide and array); eight clinical measurements (age, sex, years of education, illness duration, HDRS score, YMRS score, remission status, and TICV); and the CTs of 75 brain regions (data not shown). Genes linked to CpG sites in the lightgreen module were annotated with gene sets related to neuronal apoptosis and synaptic transmission (Supplementary Table S8).

## Discussion

This study provides the first comprehensive analysis to identify BD-specific epigenetic variations and their correlations with structural changes in the brain. Using strict criteria, we identified 156 DMPs and seven DMRs associated with either muscle hypertrophy or neuronal structure and function. Among them, 13 DMPs were significantly correlated with the CTs of the right postcentral gyrus and pars triangularis in patients with BD. These findings contribute significantly to a deeper understanding of the pathophysiology of BD and highlight genes that warrant further analysis.

Among the top 20 DMPs, the most significant CpG site was associated with the *WNT6* gene, which encodes a hydrophobic glycoprotein belonging to the Wingless/integrase 1 (WNT) family (Yuan et al., 2013). WNT signaling is known to play a crucial role in the development, function, and structure of the central nervous system and is associated with BD, schizophrenia, and Alzheimer’s disease (AD) (Berwick & Harvey, 2014; Folke, Pakkenberg, & Brudek, 2019; Hoseth et al., 2018; Inestrosa & Arenas, 2010). A CpG site associated with the *PLEC1* gene showed a positive correlation with the right postcentral gyrus CT. This gene encodes plectin, a well-known cytoskeletal linker protein that is abundant in the brain, muscles, and epithelial cells. Changes in plectin expression are known to negatively impact the structure and function of the blood–brain barrier and the pial surface (Errante, Wiche, & Shaw, 1994; Lie et al., 1998). Martins-de-Souza *et al*. have reported increased plectin expression in the brains of patients with schizophrenia (Martins-de-Souza et al., 2009). Thus, our results suggest that epigenetic variation in the *PLEC1* gene may mediate structural changes in the brains of patients with BD. The *BRUNOL4* gene, also known as *CELF4*, is abundantly expressed in the brain and plays a significant role in alternative splicing (Cahoy et al., 2008). Salamon *et al*. have reported that the target mRNA of the *BRUNOL4* gene is associated with genes that affect neurodevelopmental processes negatively (Salamon et al., 2023). Additionally, the *BRUNOL4* gene has been reported to be associated with seizures and autism spectrum disorder (Gilling et al., 2008; Halgren et al., 2012). The *SLC38A4* gene, which was associated with the most significant DMR, encodes a sodium-dependent neutral amino acid transporter belonging to the solute carrier (SLC) protein family. The instability of SLC proteins is known to be associated with various psychiatric and neurodegenerative disorders (e.g. depression, post-traumatic stress disorder [PTSD], Parkinson’s disease, and AD) (Aykac & Sehirli, 2020). In summary, epigenetic variations in these genes could play a crucial role in the pathophysiology of BD.

The functional enrichment analysis revealed biological processes related to neuronal structure and function (e.g. ‘regulation of delayed rectifier potassium channel activity’, ‘myelination in peripheral nervous system’, ‘regulation of Arp2/3 complex-mediated actin nucleation’, and ‘peripheral nervous system axon ensheathment’), and muscle hypertrophy (e.g. ‘regulation of muscle adaptation’, and ‘positive regulation of cardiac muscle hypertrophy’). Dysfunction of neural circuits and myelination is associated with psychiatric disorders, including BD and schizophrenia (Kim et al., 2013; Stern et al., 2020; Valdés-Tovar et al., 2022). Additionally, retinol metabolism has been identified as an important pathway that causes psychiatric side effects (Bremner, Shearer, & McCaffery, 2012). Excessive exposure to vitamin A, which exists in retinoic acid, can result in various neurological and psychological symptoms, including depression, fatigue, irritability, and decreased interest (Bremner & McCaffery, 2008).

The CNA analysis identified 15 focal amplifications and 14 focal deletions. We observed recurrent focal amplifications and deletions in the HLA region (6p21.33, 6p22.1, and 6p21.32), which contains genes associated with immune responses. Moreover, genes in the HLA region play important roles in synaptic development and plasticity (Elmer & McAllister, 2012; Huh et al., 2000). The 1p13.3 focal amplification region contains the glutathione S-transferase family (*GSTM1*, *GSTM2*, *GSTM5*), which is involved in the synthesis of glutathione and protection against oxidative stress (Do et al., 2009). These genes have been reported to be associated with schizophrenia, PTSD, and BD (Chaumette et al., 2019; Rezaei, Saadat, & Saadat, 2012; Tylee et al., 2015). Overlapping focal amplification and deletion regions were observed at 8p23.1, which contains the *FLJ10661* gene and is known to be part of a potential neuropsychiatric hub (Tabarés-Seisdedos & Rubenstein, 2009). Duplication and deletion syndromes involving this region share several common phenotypes (e.g. developmental delays and heart defects) (Barber et al., 2010; Montenegro et al., 2023). Thus, the functions associated with this region should be investigated further.

We observed a significant reduction in CT across a broad area of the prefrontal cortex in patients with BD (e.g. the frontomarginal, transverse frontopolar, straight, and superior and middle frontal gyri). Consistent with previous findings, we observed evidence of a strong association between structural variations in the prefrontal cortex and the pathophysiology of BD; thinning of the somatosensory cortex, including the postcentral gyrus, was observed (Foland-Ross et al., 2011; Hanford et al., 2016; Lyoo et al., 2006). The postcentral gyrus processes sensory information from the body and senses internal bodily responses to emotional contexts and is known to be deeply involved in the recognition of facial emotions, affective-related activity, and emotion processing (Chen et al., 2008; Minuzzi et al., 2018). A recent structural neuroimaging study by the ENIGMA Consortium using data from 2,447 patients with BD and 4,056 HCs reported significant cortical thinning in the bilateral postcentral gyri in patients with BD compared to HCs (Hibar et al., 2018). Several studies have reported functional connectivity changes in the postcentral gyrus in BD, and a recent large-scale meta-analysis of resting-state functional MRI data from 1842 patients with BD and 2190 HCs observed that the BD group showed significantly decreased functional activity in the left postcentral gyrus compared with the HC group (G. Chen et al., 2022; Liu et al., 2012; Minuzzi et al., 2018). Investigating the associations between these brain regions and epigenetic loci could provide new insights into the complex pathophysiology of BD.

We identified significant correlations between the methylation levels of 13 DMPs associated with three genes (*miR886*, *ICAM5*, and *PLEC1*) and the CTs of the right postcentral gyrus and pars triangularis. Then, 10 of the 13 DMPs were linked to the miR886 gene and exhibited a negative correlation with the CT of the right postcentral gyrus. Mechanistically, the nc886/miR886 (vtRNA2-1) locus emerging from our analysis may influence CT by modulating intracellular stress signaling and synaptic integrity. nc886 encodes a small vault RNA that negatively regulates the protein kinase R (PKR) pathway, an important mediator of the cellular stress response (Calderon & Conn, 2017). When nc886 is expressed, it binds to PKR and prevents aberrant kinase activation (Calderon & Conn, 2017). Hypermethylation of the nc886 promoter in BD (as observed in our data) likely silences this regulatory RNA, leading to disinhibition of PKR activity. Elevated PKR, in turn, phosphorylates eIF2α and broadly reduces protein synthesis in neurons (Calderon & Conn, 2017), including the synthesis of proteins crucial for dendritic maintenance and synaptic plasticity. Consistent with this model, mice lacking PKR exhibit enhanced synaptic plasticity and memory, whereas PKR overactivation impairs synaptic function (Gal-Ben-Ari, Barrera, Ehrlich, & Rosenblum, 2018; Zhu et al., 2011). Chronic PKR activation can also trigger proapoptotic pathways and neuroinflammation (Lee, Kunkeaw, & Lee, 2020). Therefore, in the brains of patients with BD, nc886 silencing could lead to excess PKR-mediated signaling, driving subtle neuronal loss, synaptic regression, and cortical thinning in regions like the postcentral gyrus. Supporting this, our co-methylation module analysis identified nc886-associated CpGs alongside genes involved in neuronal apoptosis and synaptic transmission, suggesting a convergent effect on these neurobiological processes. Furthermore, nc886 resides in a uniquely ‘tunable’ imprinted region subject to epigenetic polymorphism (Carpenter et al., 2018; Romanelli et al., 2014). The nc886 allele from the mother is often methylated and inactive in the majority of individuals (Romanelli et al., 2014), and importantly, this imprinting status can be influenced by maternal environment and can persist through development (Carpenter et al., 2018). Such heritable epigenetic variability in nc886 might help explain why only the BD group (and not HCs) showed a methylation–CT correlation in our study – the effect may manifest primarily against a background of other risk factors or inherited epigenetic marks in BD. In summary, we propose that nc886 methylation may contribute to cortical thinning in BD by releasing PKR-driven stress pathways that impair synaptic maintenance and promote inflammation/apoptosis, as well as by reflecting a familial epigenetic predisposition (through its imprinting mechanism) that makes the BD brain more susceptible to these effects. This hypothesis offers a testable neurobiological context for our finding of an association between miR886 methylation and cortical structure in BD.

DMP associated with the *ICAM5* gene showed a positive correlation with the right postcentral gyrus CT. The *ICAM5* gene is highly expressed in neurons, and its proper expression is crucial for synapse formation and immune responses (Matsuno et al., 2006; Mori et al., 1987; Ning et al., 2013; Tian et al., 2008). Studies using postmortem brain tissue have consistently confirmed an association between the development of BD and chronic, low-grade brain inflammation (Giridharan et al., 2020). Thus, the *ICAM5* gene could mediate the pathophysiology of BD and brain immune responses. The *PLEC1* gene was discussed above because the same significant CpG site (cg04255391) was identified in both the DMP and neuroimaging–epigenetic analyses.

Of the 18 modules identified through co-methylation analysis, the lightgreen module showed a negative correlation with the CT of the right postcentral gyrus and contains 12 hub CpG sites associated with the upstream CpG island of the *miR886* gene. Moreover, the lightgreen module was observed to be significantly enriched in genes associated with neuronal apoptosis and synaptic transmission (*GRIK2*, *VPS54*, *VAMP2*, and *DLGAP2*). These genes, along with the *miR886* gene, could therefore be potential candidate genes relevant to structural alterations in the brains of patients with BD.

This study has several limitations. First, the potential effects of participants’ psychotropic medications on DNA methylation and brain structure must be considered, as prior work indicates that such drugs can induce epigenetic alterations and neuroanatomical changes (Chopra et al., 2021; Dubath et al., 2024). Second, the sample size of our EWAS is relatively modest, which reduces statistical power and likely captures only a fraction of the true differential methylation signals (Hesam-Shariati et al., 2022; Mirza et al., 2024; Mullins et al., 2021). As with many EWASs, replication in larger independent cohorts is needed to confirm these findings. Third, we were unable to account for all possible confounders or perform detailed subgroup analyses. Unmeasured factors – including family history of psychiatric illness, sex-specific differences, comorbid conditions, and environmental influences such as lifestyle or stress exposures – may have affected DNA methylation and brain outcomes in ways we could not assess (Hesam-Shariati et al., 2022; Hibar et al., 2018; Mirza et al., 2024). Fourth, by enrolling only patients in euthymic or depressive states and excluding those in manic or hypomanic episodes, our findings may not generalize across the full spectrum of BP. In addition, structural MRI acquisition during (hypo)mania is often practically challenging and more susceptible to motion-related artifacts, which can bias CT/volume estimates (Reuter et al., 2015). State-dependent neurobiological and epigenetic dynamics characteristic of (hypo)manic phases may yield different methylation profiles or brain-structure associations, and their omission limits the breadth of our conclusions (Abé et al., 2015; Choi et al., 2022; Ludwig & Dwivedi, 2016). These limitations highlight the need for cautious interpretation of our results and should be addressed in future studies.

In summary, we applied the first neuroimaging–epigenetic analysis in an EWAS to identify novel epigenetic loci associated with structural changes in the brain in BD. The EWAS identified 156 DMPs associated with neuronal structure and function, and muscle hypertrophy. The neuroimaging–epigenetic analysis revealed 13 DMPs associated with the right postcentral gyrus and pars triangularis CT in patients with BD. Strong correlations were observed between the right postcentral gyrus CT and 10 DMPs associated with a CpG island in the upstream region of the *miR886* gene, suggesting that epigenetic changes might play an important role in brain structural changes in patients with BD. Our findings provide deep insights into the pathophysiological mechanisms of BD. Future studies aiming to explore the molecular interactions associated with DMPs using comprehensive omics data will help to identify effective therapeutic targets for BD.

Note: BD, ‘bipolar disorder’; HC, ‘healthy control’; Δβ, ‘the average β value of patients with BD minus the average β value of HCs’; DMP, ‘differentially methylated probe’; FDR, ‘false discovery rate’; CGI, ‘CpG island’; TSS1500, ‘200–1500 bp upstream of the transcriptional start site’; TSS200, ‘0–200 bp upstream of the transcriptional start site’; 5′UTR, ‘between the transcriptional start site and the ATG start site’; 1stExon, ‘first exon’; Body, ‘region between the ATG and stop codons’; 3′UTR, ‘region between the stop codon and poly A signal’; IGR, ‘intergenic region’; Shelf, ‘2–4 kb from a CpG island’; Shore, ‘0–2 kb from a CpG island’; Opensea, ‘>4 kb from a CpG island’; Island, ‘CpG island’.

Note: BD, ‘bipolar disorder’; HC, ‘healthy control’; r, ‘Pearson’s partial correlation coefficient’; FDR, ‘false discovery rate’; R, ‘right hemisphere’.

## Supporting information

10.1017/S003329172610347X.sm001 Yang et al. supplementary material Yang et al. supplementary material

## Supplementary material

The supplementary material for this article can be found at http://doi.org/10.1017/S003329172610347X.
