# Supplementary material for: Novel epigenetic loci identified from an epigenome-wide association study underlying brain structural changes in bipolar disorder
Source: Psychol Med. 2026 Mar 5;56:e66. doi: 10.1017/S003329172610347X (PMC12969215; doi:10.1017/S003329172610347X)
Supplement: Yang et al. supplementary material [file S003329172610347Xsup001.docx]

**Novel epigenetic loci identified from an epigenome-wide association study underlying brain structural changes in bipolar disorder**

**Supplementary Methods**

**Supplementary Method 1 : Verifying genetic outlier**

To verify a single population using a data-driven approach, we conducted outlier detection based on principal component analysis (PCA) and Mahalanobis distance. The first and second components (PC1 and PC2) were used for outlier detection. Next, the Mahalanobis distances for each sample were calculated within a randomly selected sub-sample group that contains 90% of the entire dataset. Outliers were defined as samples with Mahalanobis distances that fall outside the 99.9% confidence interval of the chi-squared distribution. Samples identified as outliers in more than 200 out of 1,000 iterations were designated as genetic outliers.

**Supplementary Method 2 : DNA methylation profiling**

Peripheral blood was used to assess DNA methylation levels. Intact genomic DNA was quantified using a NanoDrop® ND-1000 UV–Vis Spectrophotometer (NanoDrop Technologies, Wilmington, DE, USA) and Quant-iT Picogreen (Invitrogen; Carlsbad, CA, USA). The Zymo EZ DNA methylation kit (Zymo Research; Irvine, CA, USA) was used to process 500 ng genomic DNA samples for bisulfite conversion, and the bisulfite-converted samples were then amplified, fragmented, purified, and hybridized to the Infinium MethylationEPIC BeadChip (Illumina Inc.; San Diego, CA, USA) according to the corresponding manufacturer protocols. Finally, the hybridized arrays were washed and scanned using the Illumina iScan system.

**Supplementary Method 3 : DNA methylation data pre-processing**

Raw signal intensity data were extracted using the Illumina Genome Studio software, and the ChAMP R package was used for quality control of the raw data (Tian et al., 2017). Samples containing more than 10% low-quality probes (with a signal detection P-value > 0.01) were excluded before downstream analysis. Additionally, probes (i) with <3 beads in >5% of samples; (ii) associated with single nucleotide polymorphisms (SNPs) (Zhou, Laird, & Shen, 2017); (iii) on a sex chromosome; (iv) annotated as having multi-hit CpG sites (Nordlund et al., 2013); (v) capturing non-CG sites; (vi) with poor mapping to GRCh38 based on Illumina Manifest; and (vii) with low reproducibility were excluded. Principal component analysis was used to identify potential outlier samples. Beta-mixture quantile normalization (BMIQ) was used to adjust for probe-type bias (Teschendorff et al., 2013). Batch effects were identified using singular value decomposition (SVD) and removed using the ComBat algorithm (Johnson, Li, & Rabinovic, 2007).

**Supplementary Method 4 : White blood cell counts**

For DNA extracted from peripheral blood, potential differences in white blood cell type distributions across participants can increase the false discovery rate (FDR) (Salas et al., 2018). The FlowSorted.Blood.EPIC R package was used to estimate the heterogeneity of six types of white blood cells (CD4+ and CD8+ T-cells, natural killer cells, B-cells, monocytes, and neutrophils) (Houseman et al., 2012; Salas et al., 2018). Linear regression was used to adjust for white blood cell heterogeneity before differential methylation analysis.

**Supplementary Method 5 : MRI data acquisition**

82 out of 90 patients with BD and 154 out of 161 HCs have brain MRI scans. T1-weighted imaging was conducted using a 3.0-Tesla TrioTM whole-body imaging system (Siemens Healthcare GmbH, Erlangen, Germany) at the Korea University MRI Center. The 3D T1-weighted magnetization-prepared rapid gradient-echo (MP-RAGE) sequence was used to acquire the T1-weighted images with the parameters previously described (repetition time = 1900 ms, echo time = 2.6 ms, field of view = 220 mm, matrix size = 256 × 256, slice thickness = 1 mm, number of coronal slices without gap = 176, voxel size = 0.86 × 0.86 × 1 mm^3^, flip angle = 16°, and number of excitations = 1) (Han et al., 2022).

**Supplementary Method 6 : Image processing**

To calculate CT, FreeSurfer provides automated procedures for reconstructing the 3D cortical surface model, segmenting the gray/white-matter boundaries, smoothing the cortical map, and parcellating the cortical regions (Dale, Fischl, & Sereno, 1999; B. Fischl, Liu, & Dale, 2001; B. Fischl, Sereno, & Dale, 1999; Bruce Fischl et al., 2002, 2004; Ségonne, Pacheco, & Fischl, 2007). According to the atlas by Destrieux et al (Destrieux, Fischl, Dale, & Halgren, 2010), the thickness of 76 cortical regions were measured using the FreeSurfer 7.2 version (Laboratory for Computational Neuroimaging, Athinoula A. Martinos Center for Biomedical Imaging, Charlestown, MA, USA; http://surfer.nmr.mgh.harvard.edu). For each cortical analysis, the cortical map was smoothed using a Gaussian kernel with a full width at half maximum of 20 mm (Han et al., 2020). In the neuroimaging analysis of the present study, the thickness of the 38 cortical gyri in each hemisphere was included. The detailed protocols for calculating and extracting CT values are described elsewhere (Gonzales et al., 2023; Han et al., 2020).

**Supplementary Method 7 : Statistical power calculation**

Considering the significant impact of DNA methylation differences on statistical power, we used the pwrEWAS R package to estimate statistical power for effect size at a given sample size (n=251; case:control ratio = 1:1.8) (Graw, Henn, Thompson, & Koestler, 2019). A 5% difference in DNA methylation reached a statistical power of 55%, while a 10% difference in DNA methylation reached a statistical power of 75% (Fig. S1).

**Supplementary Method 8 : Identifying co-methylation modules**

To further explore the co-methylation modules associated with CpG sites from the neuroimaging-epigenetic analysis, we conducted a co-methylation analysis using the weighted gene co-methylation network analysis (WGCNA) R package (Langfelder & Horvath, 2008). Patients with BD with MRI and DNA methylation data were included in the analysis to investigate the relationships between co-methylation modules and CT. Since the Illumina EPIC array is reported to have less than 0.01 technical variation in β values, DMPs with an absolute Δβ less than 0.02 were excluded from the co-methylation analysis (FDR ≤ 0.05 and |Δβ| ≥ 0.02) (Bibikova et al., 2011; Campagna et al., 2021). The adjacency matrix was derived using a soft power threshold of 5, which was determined to approximate a scale-free topology (R^2^ ≥ 0.9). The topological overlap matrix (TOM) was then calculated based on this adjacency matrix. The co-methylation modules were identified using the Dynamic Tree Cut algorithm (minimum module size = 30; minimum cut height = 0.995; deep split value = 4) (Langfelder, Zhang, & Horvath, 2008). After merging similar modules (cut height = 0.25), the correlations between the module eigengenes (MEs) and CTs of the right postcentral gyri and pars triangularis were calculated using Pearson’s correlation analysis with age, sex, and white blood cell counts as covariates. Bonferroni correction was applied to control for multiple testing (P-value ≤ 0.05/(18 modules) = 2.78 × 10^-3^).

**Supplementary Figures and Tables**


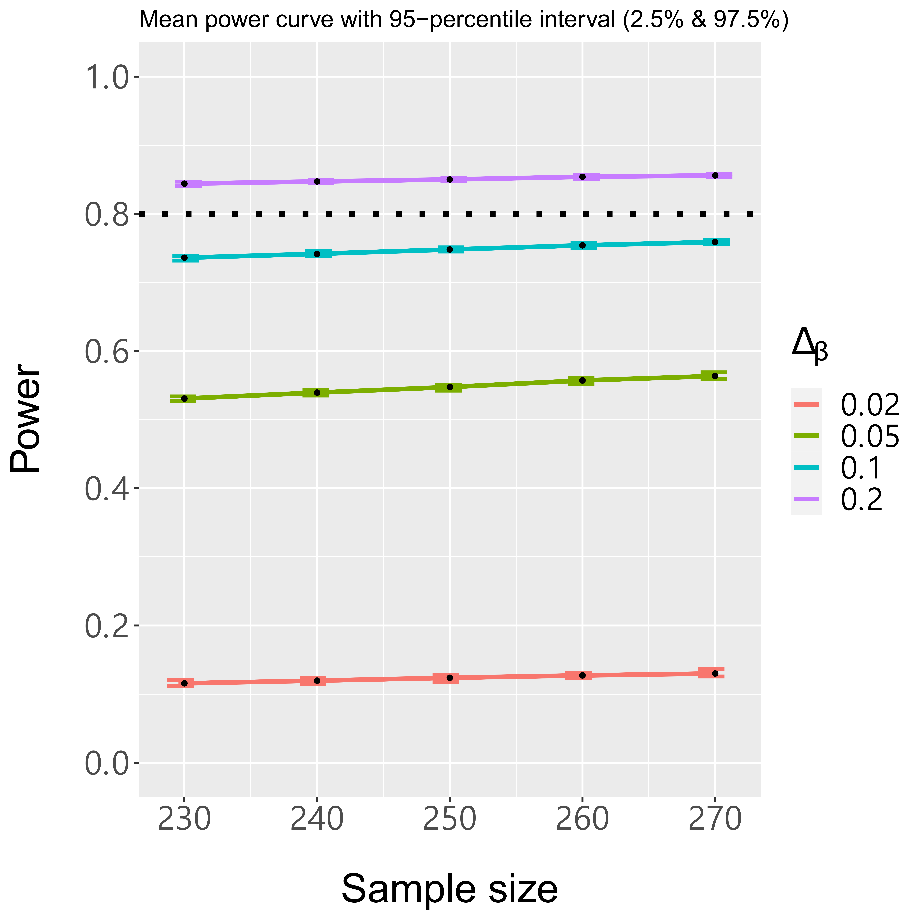


Fig. S1

Statistical power curves. X-axis: total sample size, Y-axis: statistical power. The horizontal dotted black line indicates a statistical power of 80%. Statistical power was calculated using the pwrEWAS R package (Graw et al., 2019).

BD, bipolar disorder; HC, healthy control; Δβ, average β value of patients with BD-average β value of HCs


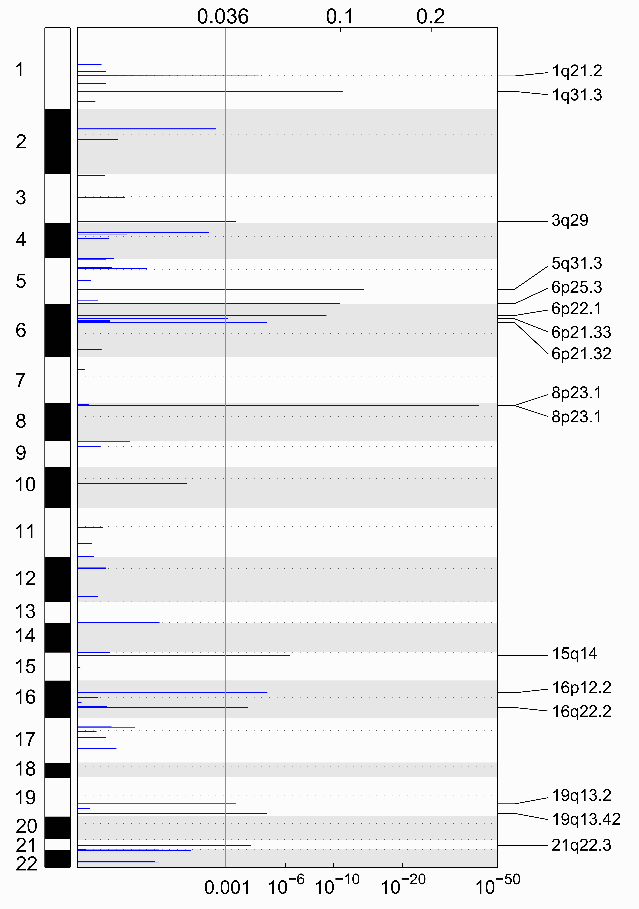

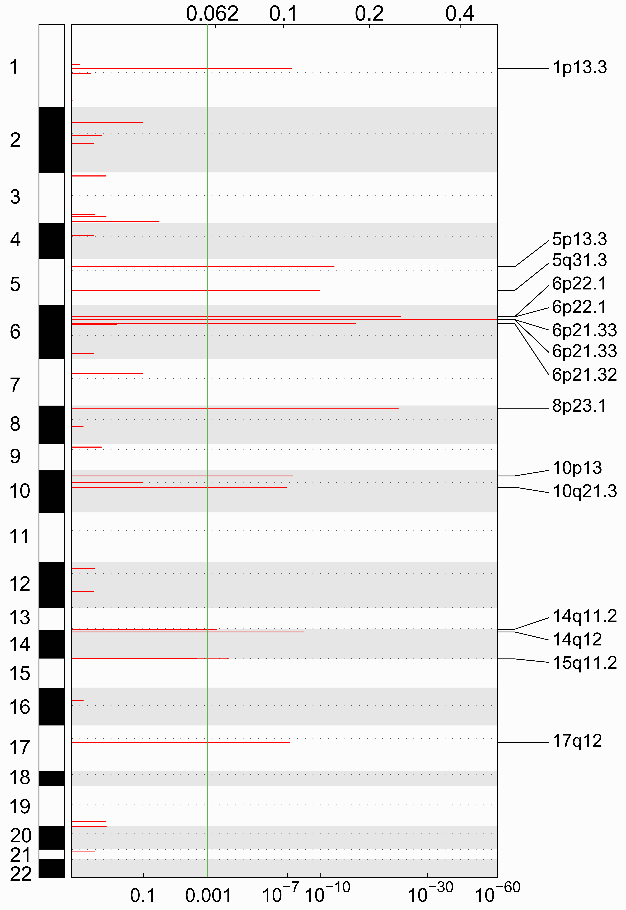


**(a)**

**(b)**

Recurrent focal (a) amplifications and (b) deletions in patients with BD are illustrated using the GISTIC2.0 algorithm (Mermel et al., 2011). Chromosome numbers are labeled on the left, and cytobands are labeled on the right side for each plot. The vertical green line indicates the cutoff FDR of 1.0 × 10^-3^.

Fig. S2

BD, bipolar disorder; HC, healthy control; FDR, false discovery rate


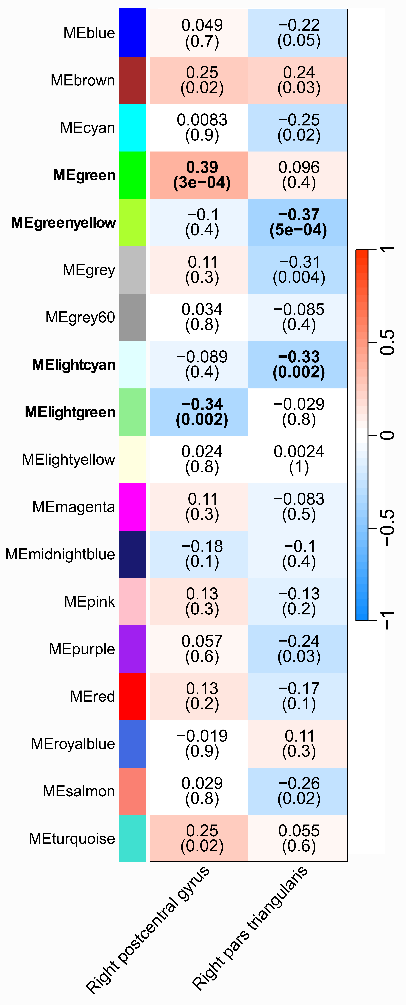


**(b)**

**(a)**

**(c)**


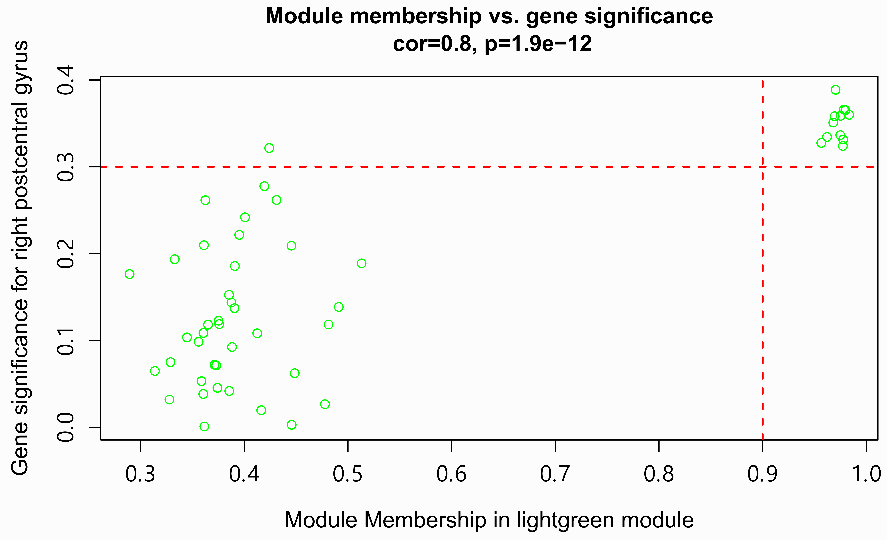

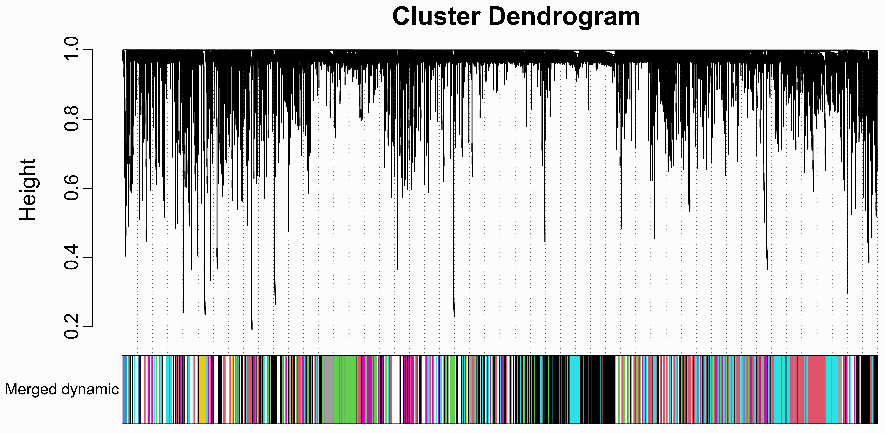


Fig. S3

(a) Cluster dendrogram based on the TOM of 11,677 CpG sites. The bottom panel shows the 18 merged co-methylation modules in patients with BD. (b) Heatmap illustrating the correlation between MEs and traits. Significant correlations and corresponding modules are highlighted in bold (P-value ≤ 2.78 × 10^-3^). (c) Scatter plot of module membership (lightgreen module) and gene significance (cortical thickness of the right postcentral gyrus). Criteria for hub CpG sites are represented as dashed red lines (|module membership| ≥ 0.9; |gene significance| ≥ 0.3). Pearson’s correlation coefficient (cor) and P-value (p) are shown.

BD, bipolar disorder; TOM, topological overlap matrix; ME, module eigengene

**Table S1. DMPs obtained by differential methylation analysis of patients with BD (n = 90) and HCs (n = 161) at the probe level (FDR ≤ 0.05, |Δβ| ≥ 0.07)**

| **CpG site** | **Δβ** | **P-value** | **FDR^a^** | **CHR** | **Position^b^** | **Gene** | **Functional region^c^** | **CGI region^d^** | **SNP id** | **SNP distance** |
| --- | --- | --- | --- | --- | --- | --- | --- | --- | --- | --- |
| cg13903421 | 0.082 | 1.94E-16 | 1.34E-11 | chr2 | 218873992 | *WNT6* | 3'UTR | Island | rs183324367 | 38 |
| cg04255391 | 0.076 | 3.41E-16 | 1.78E-11 | chr8 | 143934229 | *PLEC1* | Body | Shore | rs568424450;rs535950885 | 49;1 |
| cg13392957 | 0.087 | 3.34E-14 | 3.93E-10 | chr1 | 148310171 |  | IGR | Island | rs61810610 | 19 |
| cg03356492 | 0.081 | 5.44E-14 | 5.30E-10 | chr18 | 37482575 | *BRUNOL4* | Body | Shelf | rs542872322 | 1 |
| cg01938825 | 0.106 | 1.57E-13 | 9.56E-10 | chr7 | 1524072 |  | IGR | opensea | rs572430302;rs4725180;rs561432505 | 0;24;28 |
| cg03242265 | 0.103 | 1.61E-13 | 9.70E-10 | chr1 | 148310364 |  | IGR | Island | rs587753329 | 42 |
| cg13067553 | 0.084 | 4.51E-13 | 1.99E-09 | chr1 | 148310344 |  | IGR | Island | rs181317539;rs587714000 | 0;13 |
| cg03249723 | 0.181 | 6.10E-13 | 2.49E-09 | chr9 | 96117775 | *LOC158434* | TSS1500 | opensea | rs183894534;rs528029820;rs73654855;rs567982365 | 2;9;10;44 |
| cg23206461 | 0.105 | 2.29E-12 | 6.44E-09 | chr1 | 148310008 |  | IGR | Island |  |  |
| cg06312072 | 0.077 | 2.43E-12 | 6.69E-09 | chr12 | 98893351 | *ANKS1B* | Body | Shore | rs562054176;rs529330113;rs184323285 | 15;21;36 |
| cg18140268 | 0.107 | 7.56E-12 | 1.53E-08 | chr1 | 148309719 |  | IGR | Shore | rs587674164;rs587703477 | 29;48 |
| cg05833946 | 0.077 | 2.15E-11 | 3.21E-08 | chr8 | 1899833 | *ARHGEF10* | Body | opensea | rs574109000;rs542737634;rs556107402;rs576038378 | 51;47;42;22 |
| cg21068178 | 0.072 | 3.51E-11 | 4.47E-08 | chr19 | 15130012 |  | IGR | opensea | rs547730042 | 7 |
| cg00020172 | 0.141 | 5.92E-11 | 6.43E-08 | chr6 | 31082596 |  | IGR | opensea |  |  |
| cg02331830 | 0.079 | 7.35E-11 | 7.61E-08 | chr8 | 143934120 | *PLEC1* | Body | Shore | rs563926955;rs531197745;rs552889311;rs571191471;rs528491535 | 0;1;8;31;50 |
| cg17984267 | -0.073 | 9.07E-11 | 8.79E-08 | chr4 | 185007740 |  | IGR | Shelf | rs574958137;rs540519004 | 13;1 |
| cg07283849 | -0.089 | 2.68E-10 | 1.91E-07 | chr7 | 97771750 |  | IGR | opensea | rs541308383;rs563985858;rs1848859;rs186521113;rs563061028 | 0;13;25;30;31 |
| cg10604476 | 0.103 | 3.09E-10 | 2.12E-07 | chr19 | 10293232 | *ICAM5* | Body | Island | rs140395213;rs201216338 | 42;32 |
| cg21428710 | 0.075 | 4.47E-10 | 2.75E-07 | chr12 | 46826014 | *SLC38A4* | TSS200 | opensea | rs73104771;rs565033033 | 36;39 |
| cg16597280 | 0.097 | 1.24E-09 | 5.69E-07 | chr14 | 59965957 | *LRRC9* | Body | opensea |  |  |
| cg19459332 | 0.072 | 1.92E-09 | 7.83E-07 | chr15 | 97653017 |  | IGR | Island | rs192550307;rs541862600 | 45;19 |
| cg13801271 | 0.089 | 1.99E-09 | 8.00E-07 | chr19 | 46513791 |  | IGR | Island | rs61047870;rs182520098 | 48;46 |
| cg24191225 | 0.106 | 2.16E-09 | 8.47E-07 | chr12 | 9733228 | *CLECL1* | 1stExon | opensea | rs61913428 | 13 |
| cg11146691 | 0.082 | 4.41E-09 | 1.43E-06 | chr12 | 46825954 | *SLC38A4* | 1stExon | opensea | rs559111852;rs528361505 | 1;2 |
| cg07601320 | 0.082 | 6.04E-09 | 1.79E-06 | chr12 | 46826058 | *SLC38A4* | TSS200 | opensea | rs181506118;rs550843759 | 42;43 |
| cg12150299 | 0.072 | 7.46E-09 | 2.09E-06 | chr1 | 225866614 | *TMEM63A* | Body | opensea |  |  |
| cg15825916 | 0.092 | 1.35E-08 | 3.18E-06 | chr19 | 46513793 |  | IGR | Island | rs61047870;rs182520098 | 50;48 |
| cg04959453 | 0.071 | 1.40E-08 | 3.26E-06 | chr12 | 113797493 |  | IGR | opensea | rs16943230;rs111551642;rs112565620;rs145483402;rs538888148 | 29;16;12;2;1 |
| cg11331445 | 0.070 | 1.62E-08 | 3.62E-06 | chr12 | 46826094 | *SLC38A4* | TSS200 | opensea | rs181506118;rs550843759 | 6;7 |
| cg05107036 | -0.072 | 2.48E-08 | 4.97E-06 | chr8 | 140653661 |  | IGR | opensea | rs538666439 | 2 |
| cg15218522 | 0.080 | 3.18E-08 | 5.92E-06 | chr8 | 73168950 |  | IGR | opensea | rs189656614;rs180714613;rs559595936 | 25;20;1 |
| cg24135491 | 0.080 | 3.58E-08 | 6.46E-06 | chr17 | 4583804 | *SMTNL2* | TSS200 | Shore | rs6502800;rs192255020;rs11319311 | 35;22;1 |
| cg11793897 | 0.077 | 4.65E-08 | 7.84E-06 | chr4 | 154745316 | *LRAT* | Body | Shore | rs56983649 | 23 |
| cg17501823 | 0.075 | 5.71E-08 | 9.02E-06 | chr12 | 46826010 | *SLC38A4* | TSS200 | opensea | rs73104771;rs565033033 | 40;43 |
| cg01156747 | -0.212 | 6.62E-08 | 1.00E-05 | chr7 | 120659 |  | IGR | Island | rs369103423;rs141181987;rs11282365;rs556166041;rs553207727 | 32;31;9;2;1 |
| cg00413030 | 0.076 | 9.99E-08 | 1.35E-05 | chr7 | 159154446 |  | IGR | opensea | rs542546052;rs187306789;rs382552 | 0;1;47 |
| cg11811828 | 0.075 | 1.21E-07 | 1.56E-05 | chr6 | 31180889 |  | IGR | Shore | rs552731297;rs574112684;rs541496597;rs368199362;rs375405256 | 36;21;20;19;2 |
| cg03464017 | 0.074 | 1.24E-07 | 1.58E-05 | chr6 | 16217530 |  | IGR | opensea | rs150438608;rs138296899;rs115197806;rs557157928;rs142777794;rs546122691;rs192119150;rs77259661;rs114510481;rs561467773;rs139017673;rs552957913;rs563954710;rs149442766 | 51;48;39;34;30;28;25;23;18;17;12;11;2;1 |
| cg24630826 | 0.100 | 1.46E-07 | 1.78E-05 | chr16 | 8661219 |  | IGR | opensea | rs72529100;rs558060544;rs576647397;rs537494436;rs1620139 | 11;23;25;27;45 |
| cg06737308 | 0.082 | 1.56E-07 | 1.87E-05 | chr4 | 184100361 | *ENPP6* | Body | Shelf | rs576855728 | 6 |
| cg14827090 | 0.072 | 1.61E-07 | 1.92E-05 | chr14 | 100525087 | *WDR25* | Body | opensea | rs143044757 | 36 |
| cg00485540 | 0.080 | 1.64E-07 | 1.94E-05 | chr1 | 204711908 |  | IGR | opensea | rs145876195;rs6696899;rs74138541;rs10900424 | 47;41;29;28 |
| cg15780967 | 0.105 | 2.42E-07 | 2.57E-05 | chr14 | 63467306 | *PPP2R5E* | Body | opensea | rs577475178;rs138695877;rs559969823 | 46;31;1 |
| cg08383695 | 0.073 | 2.52E-07 | 2.65E-05 | chr6 | 10886113 | *SYCP2L* | TSS1500 | Shore | rs35084918 | 28 |
| cg22762992 | 0.071 | 2.87E-07 | 2.91E-05 | chr2 | 117858801 |  | IGR | Shore |  |  |
| cg20133046 | 0.074 | 3.30E-07 | 3.21E-05 | chr6 | 16217594 |  | IGR | opensea | rs114475105;rs144833460;rs537595780;rs183823372;rs9477054;rs539305305;rs552863457 | 47;43;31;30;29;25;1 |
| cg05929471 | -0.075 | 3.75E-07 | 3.53E-05 | chr1 | 227270798 | *CDC42BPA* | Body | opensea | rs555145479 | 1 |
| cg03651054 | -0.155 | 3.77E-07 | 3.54E-05 | chr13 | 49620507 |  | IGR | opensea | rs558730880 | 44 |
| cg09087222 | 0.071 | 3.90E-07 | 3.62E-05 | chr8 | 11862854 | *CTSB* | 5'UTR | opensea |  |  |
| cg15964514 | -0.070 | 4.99E-07 | 4.33E-05 | chr10 | 4733607 |  | IGR | opensea | rs115962707;rs186545868;rs10751983;rs536403382 | 18;20;27;45 |
| cg21641816 | -0.076 | 6.18E-07 | 5.04E-05 | chr10 | 4733533 |  | IGR | opensea | rs530399499;rs570023684;rs538682417 | 33;22;20 |
| cg16781264 | 0.126 | 6.69E-07 | 5.32E-05 | chr2 | 100471113 | *NMS* | Body | opensea | rs549387021;rs17024368 | 0;22 |
| cg27360277 | -0.072 | 6.83E-07 | 5.40E-05 | chr21 | 38925742 | *LOC400867* | Body | opensea | rs577064845;rs545879336 | 9;44 |
| cg06106484 | 0.089 | 7.26E-07 | 5.63E-05 | chr8 | 33127771 |  | IGR | opensea |  |  |
| cg02122327 | -0.150 | 1.45E-06 | 9.29E-05 | chr13 | 49620186 |  | IGR | opensea | rs141968586;rs548207551 | 21;31 |
| cg20395744 | 0.093 | 1.71E-06 | 1.05E-04 | chr12 | 115639867 |  | IGR | opensea |  |  |
| cg04143156 | 0.072 | 1.88E-06 | 1.12E-04 | chr4 | 152847098 | *ARFIP1* | Body | opensea | rs188883622;rs148052544 | 2;1 |
| cg12759523 | 0.071 | 1.98E-06 | 1.17E-04 | chr15 | 22949566 | *CYFIP1* | 5'UTR | opensea | rs574683493 | 1 |
| cg06536614 | -0.123 | 1.99E-06 | 1.17E-04 | chr5 | 136080692 | *miR886* | TSS200 | Island | rs577302824;rs533771522 | 7;13 |
| cg08977827 | 0.084 | 2.18E-06 | 1.25E-04 | chr10 | 5261949 |  | IGR | opensea | rs570564036;rs534436354 | 30;2 |
| cg12896271 | 0.074 | 2.63E-06 | 1.43E-04 | chr4 | 118591701 | *LOC729218* | TSS200 | opensea | rs531302012;rs551002434;rs570867932;rs533837210;rs553620012;rs12649214 | 50;35;32;23;13;4 |
| cg13375589 | 0.086 | 3.34E-06 | 1.70E-04 | chr17 | 4583830 | *SMTNL2* | TSS200 | Shore | rs192255020;rs11319311;rs73329999 | 48;27;9 |
| cg07136909 | 0.103 | 3.39E-06 | 1.72E-04 | chr11 | 71567848 |  | IGR | opensea | rs545411445;rs9651752 | 12;21 |
| cg08779649 | -0.171 | 4.38E-06 | 2.06E-04 | chr13 | 49620418 |  | IGR | opensea |  |  |
| cg11608150 | -0.102 | 5.73E-06 | 2.50E-04 | chr5 | 136080259 |  | IGR | Shore | rs375721444 | 1 |
| cg26896946 | -0.113 | 5.89E-06 | 2.55E-04 | chr5 | 136080716 | *miR886* | TSS200 | Island | rs559858088;rs527487348 | 45;51 |
| cg24088508 | -0.132 | 6.31E-06 | 2.68E-04 | chr1 | 37690790 | *C1orf109* | TSS1500 | Shore | rs542589108;rs678511 | 40;37 |
| cg25340688 | -0.120 | 7.41E-06 | 3.01E-04 | chr5 | 136080709 | *miR886* | TSS200 | Island |  |  |
| cg10066109 | -0.083 | 7.73E-06 | 3.10E-04 | chr8 | 106434919 | *OXR1* | Body | opensea | rs144253103;rs558516130 | 47;49 |
| cg08206881 | 0.089 | 9.16E-06 | 3.50E-04 | chr2 | 12177231 |  | IGR | opensea | rs13421453;rs530371131;rs545591065 | 16;32;46 |
| cg16836250 | -0.071 | 1.18E-05 | 4.20E-04 | chr11 | 87513779 |  | IGR | opensea | rs187375074;rs569679032;rs538954161 | 51;49;2 |
| cg06809965 | 0.089 | 1.19E-05 | 4.24E-04 | chr14 | 69603616 |  | IGR | opensea | rs562912039;rs530781607 | 15;16 |
| cg00124993 | -0.108 | 1.26E-05 | 4.40E-04 | chr5 | 136080723 | *miR886* | TSS200 | Island | rs559858088;rs527487348 | 38;44 |
| cg04737881 | 0.087 | 1.61E-05 | 5.24E-04 | chr4 | 182139790 | *MGC45800* | Body | Shore | rs4515200 | 15 |
| cg23584176 | 0.074 | 1.63E-05 | 5.29E-04 | chr6 | 29397301 | *OR12D2* | 1stExon | opensea | rs371147818;rs36211074;rs535902260;rs200560011;rs552924253;rs113268717;rs201460005 | 47;46;42;16;8;3;1 |
| cg12439472 | -0.076 | 1.82E-05 | 5.73E-04 | chr13 | 42991263 | *EPSTI1* | Body | Shore | rs569487973 | 25 |
| cg10553204 | 0.075 | 2.29E-05 | 6.74E-04 | chr2 | 20671435 | *GDF7* | 3'UTR | Island | rs140907072;rs4666457 | 27;7 |
| cg08745965 | -0.086 | 2.64E-05 | 7.47E-04 | chr5 | 136080840 | *miR886* | TSS1500 | Shore | rs71589303;rs9327740 | 7;18 |
| cg19097407 | 0.076 | 2.74E-05 | 7.67E-04 | chr9 | 36154753 | *GLIPR2* | Body | opensea |  |  |
| cg13835168 | 0.125 | 2.87E-05 | 7.93E-04 | chr6 | 29680979 |  | IGR | opensea | rs369674623;rs146035947;rs416560 | 0;16;29 |
| cg04481923 | -0.103 | 2.91E-05 | 8.02E-04 | chr5 | 136080516 | *miR886* | Body | Island | rs538710441;rs553551499;rs572325590 | 38;18;2 |
| cg08041448 | 0.100 | 3.31E-05 | 8.78E-04 | chr6 | 29681124 |  | IGR | opensea | rs2747432 | 8 |
| cg23505604 | 0.073 | 3.49E-05 | 9.12E-04 | chr1 | 94275743 |  | IGR | opensea | rs529984537;rs549848035;rs187292485 | 5;41;43 |
| cg17876578 | 0.084 | 3.59E-05 | 9.30E-04 | chr6 | 291859 | *DUSP22* | TSS200 | Shore | rs117996017 | 28 |
| cg16339550 | 0.088 | 4.70E-05 | 1.13E-03 | chr3 | 105376739 | *ALCAM* | Body | opensea | rs7618632;rs532324585;rs6771935 | 10;18;29 |
| cg10939912 | 0.106 | 4.77E-05 | 1.14E-03 | chr21 | 42738312 | *PDE9A* | 5'UTR | opensea | rs535813580;rs554659014;rs75905062;rs58923164;rs564974315;rs139578830 | 0;6;25;29;42;51 |
| cg08322244 | 0.074 | 5.50E-05 | 1.26E-03 | chr17 | 8163351 | *VAMP2* | TSS1500 | Shore |  |  |
| cg03641585 | 0.081 | 5.57E-05 | 1.27E-03 | chr17 | 10858083 |  | IGR | opensea |  |  |
| cg06478886 | -0.070 | 5.93E-05 | 1.33E-03 | chr5 | 136080340 |  | IGR | Shore | rs564360394 | 46 |
| cg18678645 | -0.093 | 5.99E-05 | 1.34E-03 | chr5 | 136080642 | *miR886* | TSS200 | Island | rs554848608;rs200849372;rs576316967 | 26;15;12 |
| cg16524778 | -0.087 | 7.17E-05 | 1.52E-03 | chr7 | 52501058 |  | IGR | opensea | rs531721591;rs12533603;rs139091698 | 24;41;44 |
| cg07332563 | 0.081 | 7.57E-05 | 1.58E-03 | chr6 | 291687 | *DUSP22* | TSS1500 | Shore | rs536605667 | 1 |
| cg14509133 | -0.087 | 7.64E-05 | 1.59E-03 | chr22 | 46587883 |  | IGR | opensea | rs5767276;rs80109778;rs533797227;rs147300858;rs536098355;rs539441682 | 37;34;34;30;29;2 |
| cg17256657 | -0.078 | 8.45E-05 | 1.71E-03 | chr18 | 79825418 |  | IGR | Shore | rs184747835 | 37 |
| cg06277607 | -0.085 | 8.56E-05 | 1.73E-03 | chr13 | 102854827 | *BIVM-ERCC5* | Body | opensea | rs561686751;rs529109890;rs550862188 | 16;32;45 |
| cg03028786 | -0.142 | 9.20E-05 | 1.82E-03 | chr2 | 224032708 | *SERPINE2* | 5'UTR | opensea | rs11312232;rs529472523 | 0;0 |
| cg14780466 | 0.073 | 9.79E-05 | 1.90E-03 | chr2 | 20671052 | *GDF7* | Body | Island | rs369980607;rs542280550 | 36;16 |
| cg01516881 | 0.092 | 1.08E-04 | 2.05E-03 | chr6 | 292596 | *DUSP22* | Body | Island | rs531907487;rs548791403 | 14;12 |
| cg10806175 | -0.078 | 1.72E-04 | 2.86E-03 | chr4 | 83020319 |  | IGR | opensea |  |  |
| cg05481257 | 0.073 | 1.74E-04 | 2.87E-03 | chr2 | 20670451 | *GDF7* | Body | Island | rs201215853;rs201812180 | 20;1 |
| cg20581874 | -0.072 | 1.88E-04 | 3.03E-03 | chr14 | 20723595 |  | IGR | Island | rs558621327;rs201500229;rs576726066 | 5;31;37 |
| cg11037025 | -0.097 | 1.91E-04 | 3.07E-03 | chr4 | 55894050 | *EXOC1* | Body | opensea | rs564835829;rs533545059 | 51;1 |
| cg08291996 | 0.074 | 2.11E-04 | 3.29E-03 | chr19 | 58279793 | *ZNF8* | Body | Shore | rs576638345;rs545584643 | 24;30 |
| cg19654323 | 0.073 | 2.12E-04 | 3.31E-03 | chr10 | 133608949 |  | IGR | opensea | rs181045574;rs4335491;rs144937560;rs111829120;rs556347017 | 1;7;21;46;49 |
| cg06068545 | 0.124 | 2.58E-04 | 3.80E-03 | chr1 | 214362645 | *PTPN14* | Body | opensea |  |  |
| cg23540632 | 0.070 | 3.00E-04 | 4.22E-03 | chr18 | 69586212 | *DOK6* | Body | opensea | rs4891753;rs78344943;rs551992048 | 44;24;15 |
| cg14935948 | 0.072 | 3.28E-04 | 4.49E-03 | chr13 | 106161375 |  | IGR | opensea | rs186719911 | 0 |
| cg10687131 | 0.084 | 3.51E-04 | 4.71E-03 | chr2 | 20671242 | *GDF7* | Body | Island |  |  |
| cg23008279 | 0.077 | 3.83E-04 | 5.02E-03 | chr12 | 31715446 | *AMN1* | 5'UTR | opensea | rs554755254;rs575025321 | 37;3 |
| cg20228636 | 0.098 | 4.00E-04 | 5.18E-03 | chr6 | 29680748 |  | IGR | opensea | rs141767002;rs2747431 | 10;39 |
| cg16885113 | 0.102 | 4.51E-04 | 5.65E-03 | chr6 | 29680730 |  | IGR | opensea | rs141767002 | 28 |
| cg24587835 | 0.081 | 4.81E-04 | 5.91E-03 | chr17 | 5770914 | *LOC339166* | TSS1500 | opensea | rs532956299 | 4 |
| cg19621538 | -0.085 | 5.00E-04 | 6.07E-03 | chr1 | 172973306 |  | IGR | opensea | rs71299438;rs568036903 | 30;1 |
| cg18797653 | -0.079 | 5.26E-04 | 6.30E-03 | chr5 | 136080924 | *miR886* | TSS1500 | Shore |  |  |
| cg26668828 | 0.080 | 5.61E-04 | 6.59E-03 | chr6 | 292823 | *DUSP22* | Body | Island | rs815596;rs560530509;rs577407363;rs546224187 | 10;19;23;40 |
| cg12406108 | 0.071 | 5.75E-04 | 6.71E-03 | chr13 | 114200085 |  | IGR | Island | rs7332155 | 14 |
| cg23733394 | 0.071 | 6.17E-04 | 7.05E-03 | chr1 | 904372 |  | IGR | Island |  |  |
| cg08476006 | -0.083 | 7.44E-04 | 8.06E-03 | chr6 | 88443782 |  | IGR | opensea | rs4707468;rs542899435 | 26;2 |
| cg26173986 | 0.076 | 8.12E-04 | 8.56E-03 | chr17 | 8163698 | *VAMP2* | TSS1500 | Shore | rs567001097 | 31 |
| cg19651757 | 0.077 | 8.19E-04 | 8.61E-03 | chr12 | 9943519 |  | IGR | opensea | rs570797508 | 50 |
| cg07030646 | -0.124 | 8.33E-04 | 8.72E-03 | chr3 | 30524423 |  | IGR | opensea | rs113915515;rs145142149 | 19;44 |
| cg20246743 | 0.072 | 9.00E-04 | 9.21E-03 | chr19 | 21569221 |  | IGR | opensea | rs73025877;rs541244511;rs553566245;rs73025880 | 0;17;23;34 |
| cg21548813 | 0.081 | 1.08E-03 | 1.05E-02 | chr6 | 291882 | *DUSP22* | TSS1500 | Shore | rs117996017 | 51 |
| cg19787097 | 0.078 | 1.23E-03 | 1.15E-02 | chr3 | 29335672 | *RBMS3* | Body | opensea |  |  |
| cg02513556 | 0.084 | 1.30E-03 | 1.20E-02 | chr12 | 9943303 |  | IGR | opensea | rs111696340 | 7 |
| cg21787089 | -0.120 | 1.31E-03 | 1.20E-02 | chr3 | 46442759 | *LTF* | Body | opensea | rs6441990;rs553622528;rs534434598;rs553502827;rs6441991;rs75095086 | 11;12;14;24;33;51 |
| cg15570656 | 0.103 | 1.32E-03 | 1.21E-02 | chr6 | 29680851 |  | IGR | opensea |  |  |
| cg01427108 | -0.106 | 1.38E-03 | 1.25E-02 | chr3 | 46442888 | *LTF* | Body | opensea |  |  |
| cg24299306 | 0.073 | 1.39E-03 | 1.26E-02 | chr12 | 9943553 |  | IGR | opensea | rs570797508;rs539425658 | 16;35 |
| cg04188756 | -0.070 | 1.46E-03 | 1.30E-02 | chr14 | 32693242 | *AKAP6* | Body | opensea | rs185742657 | 2 |
| cg04131969 | 0.137 | 1.52E-03 | 1.34E-02 | chr2 | 33726580 | *MYADML* | Body | Shore | rs559880668;rs3217586 | 33;9 |
| cg10786572 | 0.092 | 1.83E-03 | 1.52E-02 | chr10 | 52872562 |  | IGR | Shore | rs78528410;rs575389909;rs10508976;rs554571566;rs34346772 | 44;38;25;23;1 |
| cg24969716 | 0.076 | 1.87E-03 | 1.55E-02 | chr11 | 45385986 | *LOC399886* | Body | opensea | rs7928480;rs561822830;rs181587435 | 45;30;25 |
| cg03449857 | 0.088 | 2.16E-03 | 1.71E-02 | chr6 | 29680846 |  | IGR | opensea |  |  |
| cg18110333 | 0.090 | 2.31E-03 | 1.79E-02 | chr6 | 292329 | *DUSP22* | 1stExon | Island | rs575362233;rs115977262;rs560925034;rs530247647 | 0;13;20;27 |
| cg03395511 | 0.083 | 2.57E-03 | 1.93E-02 | chr6 | 291903 | *DUSP22* | TSS200 | Shore | rs535453941 | 6 |
| cg26708920 | -0.071 | 2.66E-03 | 1.98E-02 | chr10 | 13784317 | *FRMD4A* | Body | opensea | rs573927270;rs542813173 | 2;1 |
| cg07134666 | 0.085 | 2.77E-03 | 2.04E-02 | chr6 | 29680623 |  | IGR | opensea | rs17184563;rs558633480 | 11;13 |
| cg00588198 | 0.078 | 2.88E-03 | 2.09E-02 | chr6 | 29680675 |  | IGR | opensea | rs3129057;rs114013701 | 16;38 |
| cg15570860 | 0.122 | 2.92E-03 | 2.11E-02 | chr11 | 8965293 | *TMEM9B* | TSS1500 | Shore | rs77898208;rs10689687 | 0;1 |
| cg05840533 | -0.089 | 3.09E-03 | 2.20E-02 | chr20 | 33168137 | *C20orf70* | TSS200 | opensea | rs538746059;rs11470590;rs7262444 | 0;2;25 |
| cg09756125 | 0.072 | 3.98E-03 | 2.63E-02 | chr7 | 158458286 | *PTPRN2* | Body | Shore | rs1733165;rs139104005 | 11;1 |
| cg06378142 | 0.095 | 4.19E-03 | 2.73E-02 | chr19 | 49616376 | *PRR12* | Body | Shore |  |  |
| cg13388618 | 0.096 | 4.28E-03 | 2.77E-02 | chr2 | 237906129 | *RAMP1* | Body | opensea | rs114862906;rs535252405 | 34;33 |
| cg07504457 | 0.078 | 4.49E-03 | 2.86E-02 | chr8 | 144427027 | *VPS28* | 5'UTR | Shore | rs533868684;rs540322290;rs139513541 | 14;8;3 |
| cg06249604 | 0.085 | 4.68E-03 | 2.95E-02 | chr6 | 30071429 | *RNF39* | Body | Island | rs528904872;rs2301753;rs565673977 | 5;34;48 |
| cg03198009 | 0.070 | 4.76E-03 | 2.99E-02 | chr6 | 29680827 |  | IGR | opensea | rs114184656 | 9 |
| cg12033692 | -0.071 | 5.26E-03 | 3.20E-02 | chr17 | 76826808 |  | IGR | opensea | rs186846677 | 14 |
| cg11383134 | 0.076 | 5.33E-03 | 3.23E-02 | chr6 | 29680813 |  | IGR | opensea | rs114184656 | 23 |
| cg25824218 | 0.073 | 5.47E-03 | 3.29E-02 | chr12 | 24951864 |  | IGR | Shelf | rs547209851;rs10219508;rs536137685 | 12;19;39 |
| cg07952421 | 0.090 | 5.67E-03 | 3.37E-02 | chr4 | 68569883 | *UGT2B15* | TSS1500 | opensea |  |  |
| cg13840476 | 0.073 | 6.96E-03 | 3.89E-02 | chr7 | 47803151 | *PKD1L1* | Body | opensea | rs3832463;rs559248483;rs200294826 | 1;18;47 |
| cg02095219 | 0.088 | 6.97E-03 | 3.89E-02 | chr17 | 59899362 | *RPS6KB1* | 5'UTR | opensea | rs540362289;rs565325930 | 0;45 |
| cg19636627 | 0.079 | 7.52E-03 | 4.11E-02 | chr6 | 29681307 |  | IGR | opensea | rs3131848;rs3131847 | 9;21 |
| cg08986950 | -0.087 | 7.64E-03 | 4.15E-02 | chr5 | 96784608 | *ERAP1* | Body | opensea | rs561489297;rs138522940 | 8;24 |
| cg24100841 | 0.081 | 9.52E-03 | 4.84E-02 | chr6 | 29681247 |  | IGR | opensea | rs112470363;rs3129054 | 25;32 |

a: False discovery rate (FDR) (≤ 0.05)

b: UCSC GRCh38/hg38

c: CpGs located in functional genomic regions, TSS1500, 200–1500 bases upstream of the transcriptional start site; TSS200, 0–200 bases upstream of the transcriptional start site; 5′UTR, region between the transcriptional start site and the ATG start site; 1stExon, first exon; Body, region between the ATG and stop codon; IGR, intergenic region

d: CpGs located on CpG islands: shelf, 2–4 kb from a CpG island; shore, 0–2 kb from a CpG island; OpenSea, >4 kb from a CpG island; Island, CpG island.

DMP, differentially methylated probe; BD, bipolar disorder; HC, healthy control; Δβ (delta-beta), the average beta value of patients with BD minus the average beta value of HCs; SNP, single nucleotide polymorphism; CHR, chromosome

**Table S2. DMRs obtained from the differential methylation analysis of patients with BD (n = 90) and HCs (n = 161) at the region level (Stouffer ≤ 0.05, |mean Δβ| ≥ 0.05)**

| **CHR** | **Start^a^** | **End** | **Number of probes^b^** | **min_smoothed_FDR** | **Stouffer** | **Max Δβ** | **Mean Δβ** | **Gene** |
| --- | --- | --- | --- | --- | --- | --- | --- | --- |
| chr12 | 46825843 | 46826414 | 12 | 2.40E-58 | 6.54E-51 | 0.082 | 0.054 | *SLC38A4* |
| chr1 | 148309104 | 148310364 | 8 | 4.84E-29 | 6.06E-49 | 0.107 | 0.070 |  |
| chr5 | 136080073 | 136080924 | 12 | 2.54E-14 | 1.05E-19 | -0.123 | -0.090 | *miR886* |
| chr2 | 20670327 | 20671641 | 9 | 6.83E-10 | 6.83E-18 | 0.084 | 0.067 | *GDF7* |
| chr6 | 29680384 | 29681307 | 22 | 1.27E-12 | 3.63E-16 | 0.125 | 0.070 | *ZFP57* |
| chr6 | 32583972 | 32584428 | 7 | 1.05E-08 | 3.16E-09 | 0.081 | 0.053 | *HLA-DRB1* |
| chr6 | 291859 | 292596 | 7 | 7.41E-04 | 1.45E-05 | 0.092 | 0.081 | *DUSP22* |

a: UCSC GRCh38/hg38

b: Number of CpG sites in the region.

c: the Benjamini-Hochberg (BH) approach was used (FDR ≤ 0.05).

DMR, differentially methylated region; BD, bipolar disorder; HC, healthy control; FDR, false discovery rate; Δβ (delta-beta), the average beta value of patients with BD minus the average beta value of HCs; TSS1500, 200–1500 bases upstream of the transcriptional start site; TSS200, 0–200 bases upstream of the transcriptional start site; 5′UTR, between the transcriptional start site and the ATG start site; 1stExon, first exon; body, between the ATG and stop codon; IGR, intergenic region; Shelf, 2–4 kb from a CpG island; Shore, 0–2 kb from a CpG island; OpenSea, >4 kb from a CpG island; Island, CpG island; CHR, chromosome

**Table S3. Functional enrichment analysis for genes associated with EWAS results (156 DMPs) (nominal P-value ≤ 0.05, gene count ≥ 2)**

| **Category** | | **ID** | **Description** | **Gene count^a^** | **P-value** |
| --- | --- | --- | --- | --- | --- |
| Gene Ontology | Biological Process (BP) | GO:1902259 | regulation of delayed rectifier potassium channel activity | 2 | 1.71E-03 |
|  |  | GO:0043502 | regulation of muscle adaptation | 3 | 4.40E-03 |
|  |  | GO:0034315 | regulation of Arp2/3 complex-mediated actin nucleation | 2 | 4.43E-03 |
|  |  | GO:0010613 | positive regulation of cardiac muscle hypertrophy | 2 | 5.52E-03 |
|  |  | GO:0014742 | positive regulation of muscle hypertrophy | 2 | 5.69E-03 |
|  |  | GO:0051223 | regulation of protein transport | 6 | 6.48E-03 |
|  |  | GO:0022011 | myelination in peripheral nervous system | 2 | 7.65E-03 |
|  |  | GO:0032292 | peripheral nervous system axon ensheathment | 2 | 7.65E-03 |
|  |  | GO:0043500 | muscle adaptation | 3 | 7.97E-03 |
|  |  | GO:0070201 | regulation of establishment of protein localization | 6 | 8.11E-03 |
|  |  | GO:0014044 | Schwann cell development | 2 | 8.37E-03 |
|  |  | GO:0051125 | regulation of actin nucleation | 2 | 8.38E-03 |
|  |  | GO:0034314 | Arp2/3 complex-mediated actin nucleation | 2 | 9.14E-03 |
|  |  | GO:0090148 | membrane fission | 2 | 9.54E-03 |
|  |  | GO:0051602 | response to electrical stimulus | 2 | 9.55E-03 |
|  |  | GO:0014037 | Schwann cell differentiation | 2 | 1.16E-02 |
|  |  | GO:0006470 | protein dephosphorylation | 4 | 1.47E-02 |
|  |  | GO:0051049 | regulation of transport | 12 | 1.54E-02 |
|  |  | GO:0045010 | actin nucleation | 2 | 1.62E-02 |
|  |  | GO:0042552 | myelination | 3 | 1.72E-02 |
|  |  | GO:0007272 | ensheathment of neurons | 3 | 1.78E-02 |
|  |  | GO:0008366 | axon ensheathment | 3 | 1.78E-02 |
|  |  | GO:0051222 | positive regulation of protein transport | 4 | 1.80E-02 |
|  |  | GO:0070169 | positive regulation of biomineral tissue development | 2 | 1.87E-02 |
|  |  | GO:1901701 | cellular response to oxygen-containing compound | 9 | 1.90E-02 |
|  |  | GO:0110151 | positive regulation of biomineralization | 2 | 1.96E-02 |
|  |  | GO:1904951 | positive regulation of establishment of protein localization | 4 | 2.07E-02 |
|  |  | GO:0006893 | Golgi to plasma membrane transport | 2 | 2.11E-02 |
|  |  | GO:1901016 | regulation of potassium ion transmembrane transporter activity | 2 | 2.16E-02 |
|  |  | GO:0010611 | regulation of cardiac muscle hypertrophy | 2 | 2.30E-02 |
|  |  | GO:0051248 | negative regulation of protein metabolic process | 8 | 2.32E-02 |
|  |  | GO:0014743 | regulation of muscle hypertrophy | 2 | 2.45E-02 |
|  |  | GO:0009749 | response to glucose | 3 | 2.62E-02 |
|  |  | GO:0009746 | response to hexose | 3 | 2.76E-02 |
|  |  | GO:0048639 | positive regulation of developmental growth | 3 | 2.84E-02 |
|  |  | GO:0048015 | phosphatidylinositol-mediated signaling | 3 | 2.97E-02 |
|  |  | GO:0010466 | negative regulation of peptidase activity | 3 | 2.99E-02 |
|  |  | GO:0009306 | protein secretion | 4 | 3.04E-02 |
|  |  | GO:0035592 | establishment of protein localization to extracellular region | 4 | 3.05E-02 |
|  |  | GO:0034284 | response to monosaccharide | 3 | 3.09E-02 |
|  |  | GO:0048017 | inositol lipid-mediated signaling | 3 | 3.17E-02 |
|  |  | GO:0071692 | protein localization to extracellular region | 4 | 3.31E-02 |
|  |  | GO:0009617 | response to bacterium | 5 | 3.46E-02 |
|  |  | GO:0042445 | hormone metabolic process | 3 | 3.52E-02 |
|  |  | GO:0051240 | positive regulation of multicellular organismal process | 10 | 3.57E-02 |
|  |  | GO:0036465 | synaptic vesicle recycling | 2 | 3.60E-02 |
|  |  | GO:0016311 | dephosphorylation | 4 | 3.73E-02 |
|  |  | GO:0019935 | cyclic-nucleotide-mediated signaling | 2 | 3.83E-02 |
|  |  | GO:0009612 | response to mechanical stimulus | 3 | 3.96E-02 |
|  |  | GO:0032869 | cellular response to insulin stimulus | 3 | 4.07E-02 |
|  |  | GO:0055013 | cardiac muscle cell development | 2 | 4.08E-02 |
|  |  | GO:0009743 | response to carbohydrate | 3 | 4.36E-02 |
|  |  | GO:0007422 | peripheral nervous system development | 2 | 4.61E-02 |
|  |  | GO:0009607 | response to biotic stimulus | 8 | 4.66E-02 |
|  |  | GO:1901700 | response to oxygen-containing compound | 10 | 4.70E-02 |
|  |  | GO:0070167 | regulation of biomineral tissue development | 2 | 4.76E-02 |
|  |  | GO:0055006 | cardiac cell development | 2 | 4.76E-02 |
|  |  | GO:0003300 | cardiac muscle hypertrophy | 2 | 4.78E-02 |
|  |  | GO:1901379 | regulation of potassium ion transmembrane transport | 2 | 4.87E-02 |
|  |  | GO:0110149 | regulation of biomineralization | 2 | 4.94E-02 |
|  |  | GO:0014897 | striated muscle hypertrophy | 2 | 4.98E-02 |
|  |  | GO:0031032 | actomyosin structure organization | 3 | 5.00E-02 |
|  | Cellular Component (CC) | GO:0048471 | perinuclear region of cytoplasm | 9 | 1.04E-03 |
|  |  | GO:0070820 | tertiary granule | 4 | 1.24E-03 |
|  |  | GO:0030141 | secretory granule | 8 | 3.85E-03 |
|  |  | GO:1903561 | extracellular vesicle | 14 | 6.27E-03 |
|  |  | GO:0043230 | extracellular organelle | 14 | 6.30E-03 |
|  |  | GO:0065010 | extracellular membrane-bounded organelle | 14 | 6.30E-03 |
|  |  | GO:0031941 | filamentous actin | 2 | 7.47E-03 |
|  |  | GO:1904724 | tertiary granule lumen | 2 | 8.44E-03 |
|  |  | GO:0070161 | anchoring junction | 11 | 1.25E-02 |
|  |  | GO:0070062 | extracellular exosome | 13 | 1.30E-02 |
|  |  | GO:0099503 | secretory vesicle | 8 | 1.37E-02 |
|  |  | GO:0042581 | specific granule | 3 | 1.39E-02 |
|  |  | GO:0035580 | specific granule lumen | 2 | 1.45E-02 |
|  |  | GO:0042383 | sarcolemma | 3 | 1.89E-02 |
|  |  | GO:0009986 | cell surface | 7 | 2.18E-02 |
|  |  | GO:0030054 | cell junction | 13 | 4.88E-02 |
|  |  | GO:0016528 | sarcoplasm | 2 | 4.93E-02 |
|  | Molecular Function (MF) | GO:0001530 | lipopolysaccharide binding | 2 | 3.84E-03 |
|  |  | GO:0004725 | protein tyrosine phosphatase activity | 3 | 6.93E-03 |
|  |  | GO:0042578 | phosphoric ester hydrolase activity | 5 | 9.76E-03 |
|  |  | GO:0004721 | phosphoprotein phosphatase activity | 3 | 2.80E-02 |
|  |  | GO:0043621 | protein self-association | 2 | 2.91E-02 |
|  |  | GO:0005102 | signaling receptor binding | 9 | 4.03E-02 |
|  |  | GO:0008081 | phosphoric diester hydrolase activity | 2 | 4.58E-02 |
| KEGG pathway | | hsa00830 | Retinol metabolism | 2 | 8.57E-03 |

a: Number of genes associated with DMPs in term

**Table S4. Copy number alterations identified between patients with BD (n = 90) and HCs (n = 161)**

| **Cytoband** | **CHR^a^** | **Start** | **End** | **Event** | **FDR^b^** | **Number of BD subjects (N = 90)** | **Length (bp)** | **Number of genes** | **Gene^c^** |
| --- | --- | --- | --- | --- | --- | --- | --- | --- | --- |
| 1p13.3 | chr1 | 109670490 | 109713871 | CN gain | 4.17E-08 | 8 | 43381 | 3 | *GSTM1; GSTM2; GSTM5* |
| 1q21.2 | chr1 | 144394261 | 148520102 | CN loss | 3.47E-05 | 2 | 4125841 | 7 | *NBPF15; LOC388692; PPIAL4D; LOC645166; NBPF16; PPIAL4F; PPIAL4E* |
| 1q31.3 | chr1 | 196733802 | 196890798 | CN loss | 1.24E-11 | 5 | 156996 | 2 | *CFHR1; CFHR3* |
| 3q29 | chr3 | 195687105 | 195721325 | CN loss | 4.08E-04 | 3 | 34220 | 2 | *hsa-mir-570; miR570* |
| 5p13.3 | chr5 | 32100067 | 32172249 | CN gain | 2.61E-12 | 6 | 72182 | 2 | *PDZD2; GOLPH3* |
| 5q31.3 | chr5 | 141173511 | 141180289 | CN gain | 1.22E-10 | 6 | 6778 | 2 | *PCDHB8; PCDHB7* |
| 5q31.3 | chr5 | 140841780 | 140855333 | CN loss | 3.13E-14 | 3 | 13553 | 1 | *PCDHA9* |
| 6p22.1 | chr6 | 29888365 | 29942491 | CN gain | 1.43E-23 | 20 | 54126 | 3 | *HLA-A; HLA-H; HCG4B* |
| 6p22.1 | chr6 | 30071249 | 30071822 | CN gain | 3.39E-18 | 13 | 573 | 1 | *RNF39* |
| 6p22.1 | chr6 | 29889736 | 29942491 | CN loss | 5.84E-10 | 3 | 52755 | 1 | *HCG4B* |
| 6p21.33 | chr6 | 31224788 | 31271989 | CN gain | 4.21E-60 | 14 | 47201 | 1 | *HLA-C* |
| 6p21.33 | chr6 | 31481156 | 31494269 | CN gain | 5.33E-05 | 5 | 13113 | 1 | *[MICB]* |
| 6p21.33 | chr6 | 31414289 | 31498087 | CN loss | 8.82E-04 | 3 | 83798 | 2 | *HCP5; HCG26* |
| 6p21.32 | chr6 | 32476043 | 32590681 | CN gain | 2.99E-15 | 10 | 114638 | 3 | *HLA-DRB1; HLA-DRB5; HLA-DRB6* |
| 6p21.32 | chr6 | 32526141 | 32583971 | CN loss | 1.33E-05 | 2 | 57830 | 1 | *HLA-DRB6* |
| 8p23.1 | chr8 | 8026301 | 8244152 | CN gain | 3.98E-23 | 11 | 217851 | 3 | *hsa-mir-548i-3; FLJ10661; miR548I3* |
| 8p23.1 | chr8 | 8228425 | 8244598 | CN loss | 2.20E-42 | 21 | 16173 | 1 | *FLJ10661* |
| 10p13 | chr10 | 13526100 | 13529815 | CN gain | 3.36E-08 | 7 | 3715 | 1 | *[BEND7]* |
| 10q21.3 | chr10 | 68074735 | 68076844 | CN gain | 1.13E-07 | 5 | 2109 | 1 | *HERC4* |
| 14q11.2 | chr14 | 20874304 | 20955677 | CN gain | 8.68E-04 | 2 | 81373 | 3 | *RNASE2; RNASE3; ECRP* |
| 14q12 | chr14 | 24310588 | 24311683 | CN gain | 4.20E-09 | 7 | 1095 | 3 | *LTB4R; CIDEB; LTB4R2* |
| 15q11.2 | chr15 | 20282620 | 22221381 | CN gain | 2.82E-04 | 4 | 1938761 | 16 | *hsa-mir-3118-6; hsa-mir-3118-4; NBEAP1; OR4N4; HERC2P3; POTEB; LOC348120; OR4M2; OR4N3P; NF1P2; CHEK2P2; LOC646214; CXADRP2; GOLGA6L6; LOC727924; GOLGA8C* |
| 15q14 | chr15 | 34370435 | 34752610 | CN loss | 1.60E-06 | 2 | 382175 | 6 | *hsa-mir-1233-2; hsa-mir-1233-1; GOLGA8A; GOLGA8B; miR1233-1; miR1233-2* |
| 16p12.2 | chr16 | 22427857 | 22723130 | CN loss | 1.24E-05 | 4 | 295273 | 3 | *LOC641298; LOC653786; LOC100132247* |
| 16q22.2 | chr16 | 72025040 | 72085060 | CN loss | 1.18E-04 | 3 | 60020 | 2 | *HP; HPR* |
| 17q12 | chr17 | 36106873 | 36265379 | CN gain | 7.01E-08 | 5 | 158506 | 6 | *CCL3L1; CCL4L1; CCL4L2; TBC1D3B; TBC1D3C; CCL3L3* |
| 19q13.2 | chr19 | 42766853 | 43009991 | CN loss | 4.08E-04 | 4 | 243138 | 5 | *PSG1; PSG6; PSG7; PSG10P; LOC100289650* |
| 19q13.42 | chr19 | 54810865 | 54832737 | CN loss | 1.33E-05 | 3 | 21872 | 1 | *KIR3DL1* |
| 21q22.3 | chr21 | 41881296 | 41926984 | CN loss | 9.12E-05 | 0 | 45688 | 1 | *C2CD2* |
|  | | | | | | | | |  |

a: UCSC GRCh38/hg38

b: Benjamini-Hochberg (BH) approach was applied (FDR ≤ 1.0E-03).

c: The nearest gene name is indicated in brackets.

BD, bipolar disorder; HC, healthy control; FDR, false discovery rate; CN gain, copy number gain; CN loss, copy number loss; CHR, Chromosome

**Table S5. Demographic and clinical characteristics of patients with BD and HCs included in the neuroimaging analysis**

| **Characteristics** | **BD (n=82)** | **HC (n = 154)** | **P-value (t, χ^2^)** |  |
| --- | --- | --- | --- | --- |
|  |  |  |  |  |
| Age (mean ± SD) | 32.78 ± 10.96 | 38.53 ± 14.04 | <0.001^a^ (t = -3.467) |  |
| Sex (Female/Male) | 53/29 | 98/56 | 0.99^b^ (χ^2^ = 9.31E-05) |  |
| Years of education (mean ± SD) | 13.65 ± 2.38 | 15.01 ± 2.27 | <0.001^a^ (t = -4.241) |  |
| HDRS-17 score (mean ± SD) | 8.12 ± 5.58 | 1.01 ± 1.76 | <0.001^a^ (t = 11.236) |  |
| YMRS score (mean ± SD) | 1.23 ± 2.14 | NA | NA |  |
| Remission state/depressive state | 43/39 | NA | NA |  |
| Illness duration (mean ± SD) (months) | 27.20 ± 20.85 | NA | NA |  |
| TICV (mean ± SD) (cm^3^) | 1422.07 ± 159.08 | 1460.69 ± 148.90 | 0.071^a^ (t = -1.815) |  |
| Drug-treated patients (n) | 82 | NA | NA |  |
| **Medication (n)** |  |  |  |  |
| **Antidepressants** |  |  |  |  |
| SSRI | 14 | NA | NA |  |
| SNRI | 6 |  |  |  |
| NDRI | 9 |  |  |  |
| NaSSA | 2 |  |  |  |
| Others | 2 |  |  |  |
| Combination of ADs | 1 |  |  |  |
| **Mood stabilizer** |  |  |  |  |
| None | 7 |  |  |  |
| Li | 15 |  |  |  |
| AED | 55 |  |  |  |
| Combination of Li and AED | 3 |  |  |  |
| Combination of AEDs | 2 |  |  |  |
| **Antipsychotics** |  |  |  |  |
| None | 12 |  |  |  |
| AP | 44 |  |  |  |
| Combination of APs | 26 |  |  |  |

a: P-values for comparisons of age, years of education, HDRS scores, and TICV were obtained using independent t-tests.

b: P-values for sex distribution were obtained using the chi-square test.

BD, bipolar disorder; HC, healthy control; SD, standard deviation; HDRS-17, 17-item Hamilton Depression Rating Scale; YMRS, Young Mania Rating Scale; TICV, total intracranial cavity volume; SSRI, selective serotonin reuptake inhibitor; SNRI, serotonin and norepinephrine reuptake inhibitor; NDRI, norepinephrine-dopamine reuptake inhibitor; NaSSA, noradrenergic and specific serotonergic antidepressant; Combination of AD, combinations of two or more types of antidepressant; ADs, antidepressants; Li, lithium; AED, antiepileptic mood stabilizer; Combination of AEDs, combinations of two or more types of antiepileptic mood stabilizer; APs, antipsychotics; Combination of APs, combinations of two or more types of antipsychotics

**Table S6. Cortical thicknesses of 76 brain regions in the patients with BD and HC groups**

| **Cortical regions** | **BD (n = 82)** | | **HC (n = 154)** | | **F(1, 367)** | **Mean difference^a^** | **P-value** | **FDR^b^** |
| --- | --- | --- | --- | --- | --- | --- | --- | --- |
|  | **Mean** | **SD** | **Mean** | **SD** |  |  |  |  |
| L Frontomarginal gyrus | 2.272 | 0.148 | 2.353 | 0.164 | 24.506 | -0.081 | 1.43E-06 | **5.44E-05** |
| L Inferior occipital gyrus | 2.424 | 0.165 | 2.487 | 0.198 | 4.458 | -0.063 | 3.58E-02 | 5.07E-02 |
| L Paracentral lobule | 2.412 | 0.162 | 2.449 | 0.172 | 4.588 | -0.037 | 3.32E-02 | 5.05E-02 |
| L Subcentral gyrus | 2.706 | 0.161 | 2.713 | 0.176 | 3.119 | -0.007 | 7.87E-02 | 9.34E-02 |
| L Transverse frontopolar gyrus | 2.633 | 0.232 | 2.699 | 0.205 | 17.463 | -0.066 | 4.16E-05 | **1.05E-03** |
| L Anterior cingulate gyrus | 2.723 | 0.120 | 2.733 | 0.135 | 5.819 | -0.009 | 1.66E-02 | **2.75E-02** |
| L Anterior mid-cingulate gyrus | 2.627 | 0.166 | 2.642 | 0.159 | 3.174 | -0.015 | 7.61E-02 | 9.19E-02 |
| L Posterior mid-cingulate gyrus | 2.532 | 0.141 | 2.569 | 0.130 | 13.822 | -0.037 | 2.52E-04 | **2.00E-03** |
| L Dorsal posterior cingulate gyrus | 2.757 | 0.166 | 2.805 | 0.153 | 11.639 | -0.048 | 7.63E-04 | **3.36E-03** |
| L Ventral posterior cingulate gyrus | 2.547 | 0.230 | 2.571 | 0.267 | 7.829 | -0.025 | 5.58E-03 | **1.18E-02** |
| L Cuneus | 1.710 | 0.112 | 1.761 | 0.124 | 8.570 | -0.051 | 3.76E-03 | **8.93E-03** |
| L Pars opercularis | 2.788 | 0.161 | 2.808 | 0.159 | 5.467 | -0.020 | 2.02E-02 | **3.20E-02** |
| L Pars orbitalis | 2.803 | 0.196 | 2.804 | 0.207 | 1.805 | -0.002 | 1.80E-01 | 2.02E-01 |
| L Pars triangularis | 2.644 | 0.168 | 2.681 | 0.159 | 14.996 | -0.037 | 1.40E-04 | **1.59E-03** |
| L Middle frontal gyrus | 2.651 | 0.155 | 2.674 | 0.147 | 9.203 | -0.023 | 2.69E-03 | **7.17E-03** |
| L Superior frontal gyrus | 2.935 | 0.160 | 2.967 | 0.148 | 11.558 | -0.032 | 7.95E-04 | **3.36E-03** |
| L Long insular gyrus | 3.074 | 0.257 | 3.129 | 0.244 | 6.641 | -0.055 | 1.06E-02 | **1.83E-02** |
| L Short insular gyrus | 3.550 | 0.205 | 3.539 | 0.234 | 2.282 | 0.012 | 1.32E-01 | 1.52E-01 |
| L Middle occipital gyrus | 2.510 | 0.140 | 2.541 | 0.133 | 7.007 | -0.031 | 8.68E-03 | **1.61E-02** |
| L Superior occipital gyrus | 2.119 | 0.167 | 2.158 | 0.175 | 4.414 | -0.039 | 3.67E-02 | 5.07E-02 |
| L Lateral occipito-temporal gyrus | 2.753 | 0.183 | 2.822 | 0.181 | 12.841 | -0.069 | 4.14E-04 | **2.42E-03** |
| L Lingual gyrus | 1.834 | 0.135 | 1.853 | 0.128 | 2.084 | -0.019 | 1.50E-01 | 1.70E-01 |
| L Parahippocampal gyrus | 2.795 | 0.188 | 2.834 | 0.221 | 1.261 | -0.040 | 2.63E-01 | 2.77E-01 |
| L Orbital gyrus | 2.704 | 0.159 | 2.725 | 0.173 | 6.712 | -0.022 | 1.02E-02 | **1.80E-02** |
| L Angular gyrus | 2.637 | 0.142 | 2.663 | 0.145 | 8.341 | -0.026 | 4.25E-03 | **9.49E-03** |
| L Supramarginal gyrus | 2.720 | 0.167 | 2.738 | 0.164 | 10.223 | -0.019 | 1.58E-03 | **5.13E-03** |
| L Superior parietal lobule | 2.366 | 0.134 | 2.408 | 0.124 | 8.503 | -0.041 | 3.90E-03 | **8.97E-03** |
| L Postcentral gyrus | 2.168 | 0.145 | 2.215 | 0.143 | 7.458 | -0.047 | 6.80E-03 | **1.36E-02** |
| L Precentral gyrus | 2.851 | 0.180 | 2.867 | 0.214 | 1.262 | -0.017 | 2.62E-01 | 2.77E-01 |
| L Precuneus | 2.468 | 0.146 | 2.510 | 0.137 | 11.136 | -0.041 | 9.87E-04 | **3.75E-03** |
| L Straight gyrus | 2.689 | 0.171 | 2.736 | 0.199 | 14.641 | -0.046 | 1.68E-04 | **1.59E-03** |
| L Subcallosal gyrus | 2.430 | 0.217 | 2.532 | 0.199 | 11.619 | -0.102 | 7.71E-04 | **3.36E-03** |
| L Anterior transverse temporal gyrus | 2.291 | 0.230 | 2.368 | 0.246 | 9.104 | -0.077 | 2.84E-03 | **7.19E-03** |
| L Lateral superior temporal gyrus | 3.007 | 0.212 | 3.041 | 0.190 | 4.502 | -0.035 | 3.49E-02 | 5.07E-02 |
| L Planum polare | 3.232 | 0.236 | 3.309 | 0.227 | 10.699 | -0.077 | 1.24E-03 | **4.27E-03** |
| L Planum temporale | 2.502 | 0.193 | 2.548 | 0.197 | 9.409 | -0.047 | 2.42E-03 | **6.81E-03** |
| L Inferior temporal gyrus | 2.916 | 0.201 | 2.967 | 0.171 | 7.180 | -0.051 | 7.90E-03 | **1.54E-02** |
| L Middle temporal gyrus | 2.977 | 0.180 | 3.016 | 0.166 | 9.174 | -0.039 | 2.73E-03 | **7.17E-03** |
| R Frontomarginal gyrus | 2.293 | 0.168 | 2.376 | 0.195 | 15.657 | -0.083 | 1.01E-04 | **1.54E-03** |
| R Inferior occipital gyrus | 2.593 | 0.235 | 2.622 | 0.234 | 1.175 | -0.028 | 2.80E-01 | 2.91E-01 |
| R Paracentral lobule | 2.403 | 0.177 | 2.447 | 0.175 | 6.567 | -0.044 | 1.10E-02 | **1.86E-02** |
| R Subcentral gyrus | 2.707 | 0.166 | 2.688 | 0.189 | 0.057 | 0.020 | 8.11E-01 | 8.22E-01 |
| R Transverse frontopolar gyrus | 2.563 | 0.167 | 2.656 | 0.168 | 25.613 | -0.092 | 8.54E-07 | **5.44E-05** |
| R Anterior cingulate gyrus | 2.681 | 0.129 | 2.703 | 0.144 | 13.160 | -0.022 | 3.52E-04 | **2.23E-03** |
| R Anterior mid-cingulate gyrus | 2.697 | 0.150 | 2.729 | 0.138 | 10.177 | -0.033 | 1.62E-03 | **5.13E-03** |
| R Posterior mid-cingulate gyrus | 2.563 | 0.137 | 2.600 | 0.119 | 14.833 | -0.037 | 1.52E-04 | **1.59E-03** |
| R Dorsal posterior cingulate gyrus | 2.788 | 0.153 | 2.838 | 0.125 | 12.482 | -0.050 | 4.96E-04 | **2.57E-03** |
| R Ventral posterior cingulate gyrus | 2.588 | 0.210 | 2.603 | 0.210 | 4.540 | -0.015 | 3.42E-02 | 5.07E-02 |
| R Cuneus | 1.729 | 0.117 | 1.773 | 0.119 | 9.876 | -0.044 | 1.89E-03 | **5.54E-03** |
| R Pars opercularis | 2.779 | 0.165 | 2.792 | 0.146 | 4.106 | -0.012 | 4.39E-02 | 5.75E-02 |
| R Pars orbitalis | 2.795 | 0.214 | 2.809 | 0.229 | 3.375 | -0.014 | 6.75E-02 | 8.41E-02 |
| R Pars triangularis | 2.657 | 0.170 | 2.687 | 0.165 | 9.888 | -0.030 | 1.88E-03 | **5.54E-03** |
| R Middle frontal gyrus | 2.653 | 0.156 | 2.687 | 0.149 | 12.438 | -0.034 | 5.08E-04 | **2.57E-03** |
| R Superior frontal gyrus | 2.927 | 0.157 | 2.960 | 0.152 | 13.743 | -0.034 | 2.63E-04 | **2.00E-03** |
| R Long insular gyrus | 3.293 | 0.289 | 3.307 | 0.295 | 3.326 | -0.015 | 6.95E-02 | 8.52E-02 |
| R Short insular gyrus | 3.368 | 0.243 | 3.377 | 0.228 | 4.204 | -0.009 | 4.15E-02 | 5.53E-02 |
| R Middle occipital gyrus | 2.564 | 0.134 | 2.589 | 0.142 | 4.887 | -0.024 | 2.81E-02 | **4.35E-02** |
| R Superior occipital gyrus | 2.160 | 0.157 | 2.210 | 0.168 | 9.019 | -0.051 | 2.97E-03 | **7.27E-03** |
| R Lateral occipito-temporal gyrus | 2.744 | 0.178 | 2.822 | 0.178 | 13.276 | -0.078 | 3.32E-04 | **2.23E-03** |
| R Lingual gyrus | 1.872 | 0.130 | 1.917 | 0.133 | 7.058 | -0.045 | 8.45E-03 | **1.60E-02** |
| R Parahippocampal gyrus | 2.912 | 0.211 | 2.949 | 0.219 | 0.771 | -0.037 | 3.81E-01 | 3.91E-01 |
| R Orbital gyrus | 2.736 | 0.155 | 2.767 | 0.167 | 10.989 | -0.030 | 1.06E-03 | **3.85E-03** |
| R Angular gyrus | 2.624 | 0.168 | 2.651 | 0.156 | 7.652 | -0.027 | 6.13E-03 | **1.26E-02** |
| R Supramarginal gyrus | 2.733 | 0.156 | 2.741 | 0.164 | 4.031 | -0.009 | 4.58E-02 | 5.91E-02 |
| R Superior parietal lobule | 2.381 | 0.139 | 2.401 | 0.125 | 3.741 | -0.020 | 5.43E-02 | 6.88E-02 |
| R Postcentral gyrus | 2.149 | 0.177 | 2.197 | 0.153 | 5.491 | -0.048 | 2.00E-02 | **3.20E-02** |
| R Precentral gyrus | 2.830 | 0.201 | 2.817 | 0.248 | 0.005 | 0.013 | 9.42E-01 | 9.42E-01 |
| R Precuneus | 2.471 | 0.136 | 2.514 | 0.132 | 11.373 | -0.043 | 8.74E-04 | **3.50E-03** |
| R Straight gyrus | 2.645 | 0.204 | 2.705 | 0.213 | 15.651 | -0.061 | 1.01E-04 | **1.54E-03** |
| R Subcallosal gyrus | 2.554 | 0.215 | 2.614 | 0.207 | 1.477 | -0.059 | 2.25E-01 | 2.45E-01 |
| R Anterior transverse temporal gyrus | 2.359 | 0.226 | 2.427 | 0.227 | 8.253 | -0.068 | 4.45E-03 | **9.66E-03** |
| R Lateral superior temporal gyrus | 3.032 | 0.197 | 3.068 | 0.211 | 4.271 | -0.036 | 3.99E-02 | 5.41E-02 |
| R Planum polare | 3.171 | 0.234 | 3.178 | 0.227 | 1.522 | -0.008 | 2.19E-01 | 2.41E-01 |
| R Planum temporale | 2.505 | 0.183 | 2.529 | 0.185 | 4.445 | -0.024 | 3.61E-02 | 5.07E-02 |
| R Inferior temporal gyrus | 2.917 | 0.203 | 2.961 | 0.192 | 6.781 | -0.044 | 9.81E-03 | **1.78E-02** |
| R Middle temporal gyrus | 2.964 | 0.157 | 2.984 | 0.196 | 2.29E+00 | -0.020 | 1.31E-01 | 1.52E-01 |

a: (mean value of the BD group) – (mean value of the HC group)

b: Benjamini-Hochberg (BH) approach was applied (FDR ≤ 0.05). The significant FDR is represented in bold font.

BD, bipolar disorder; HC, healthy control; SD, standard deviation; FDR, false discovery rate; L, left hemisphere; R, right hemisphere

**Table S7. Light-green module CpG sites**

| **Module** | **CpG site^a^** | **CHR** | **Position^b^** | **Gene** | **Module membership^c^** | **Gene significance^d^** |
| --- | --- | --- | --- | --- | --- | --- |
| light-green | cg08352418 | 2 | 10283395 |  | 0.481 | 0.118 |
|  | cg27457290 | 2 | 64019711 | *VPS54* | 0.375 | 0.123 |
|  | cg23577671 | 2 | 121777202 |  | 0.376 | 0.119 |
|  | cg12539787 | 3 | 185721094 | *IGF2BP2-AS1* | 0.333 | 0.194 |
|  | cg00351892 | 4 | 108763387 | *ETNPPL* | 0.395 | 0.222 |
|  | **cg04515200** | 5 | 136080073 |  | **0.962** | **0.334** |
|  | **cg13581155** | 5 | 136080092 |  | **0.957** | **0.328** |
|  | **cg11608150** | 5 | 136080259 |  | **0.978** | **0.366** |
|  | **cg06478886** | 5 | 136080340 |  | **0.968** | **0.351** |
|  | **cg04481923** | 5 | 136080516 | *miR886* | **0.975** | **0.359** |
|  | **cg18678645** | 5 | 136080642 | *miR886* | **0.975** | **0.336** |
|  | **cg06536614** | 5 | 136080692 | *miR886* | **0.970** | **0.358** |
|  | **cg25340688** | 5 | 136080709 | *miR886* | **0.983** | **0.360** |
|  | **cg26896946** | 5 | 136080716 | *miR886* | **0.980** | **0.365** |
|  | **cg00124993** | 5 | 136080723 | *miR886* | **0.970** | **0.389** |
|  | **cg08745965** | 5 | 136080840 | *miR886* | **0.978** | **0.331** |
|  | **cg18797653** | 5 | 136080924 | *miR886* | **0.977** | **0.324** |
|  | cg20866694 | 6 | 27213891 |  | 0.391 | 0.137 |
|  | cg06210070 | 6 | 33117286 | *HLA-DPB2* | 0.449 | 0.062 |
|  | cg10665390 | 6 | 33117744 | *HLA-DPB2* | 0.513 | 0.189 |
|  | cg07735952 | 6 | 33117788 | *HLA-DPB2* | 0.478 | 0.027 |
|  | cg10619129 | 6 | 33117978 | *HLA-DPB2* | 0.416 | 0.020 |
|  | cg26727642 | 6 | 90413870 |  | 0.289 | 0.176 |
|  | cg23666299 | 6 | 101879044 | *GRIK2* | 0.356 | 0.099 |
|  | cg21205282 | 6 | 170244045 |  | 0.371 | 0.072 |
|  | cg24986219 | 7 | 1381502 |  | 0.385 | 0.153 |
|  | cg20947553 | 7 | 6400970 | *RAC1* | 0.446 | 0.003 |
|  | cg02796638 | 7 | 155349986 |  | 0.388 | 0.144 |
|  | cg00413030 | 7 | 159154446 |  | 0.413 | 0.108 |
|  | cg07219416 | 8 | 1658134 | *DLGAP2* | 0.359 | 0.053 |
|  | cg24895155 | 8 | 126824954 |  | 0.362 | 0.001 |
|  | cg07301298 | 9 | 94802945 | *C9orf3* | 0.362 | 0.261 |
|  | cg26649551 | 9 | 125821092 | *PBX3* | 0.314 | 0.065 |
|  | cg01285914 | 9 | 127267854 | *GARNL3* | 0.328 | 0.032 |
|  | cg01099825 | 9 | 137271662 | *COBRA1* | 0.388 | 0.092 |
|  | cg02245518 | 10 | 15800668 | *FAM188A* | 0.401 | 0.242 |
|  | cg11848931 | 10 | 24921163 | *PRTFDC1* | 0.361 | 0.210 |
|  | cg15340455 | 10 | 118627133 |  | 0.431 | 0.262 |
|  | cg26326607 | 11 | 1955040 | *MRPL23* | 0.329 | 0.075 |
|  | cg15098940 | 11 | 46930106 |  | 0.424 | **0.322** |
|  | cg12586386 | 11 | 77588760 | *AQP11* | 0.374 | 0.045 |
|  | cg12929333 | 12 | 25101219 | *LRMP* | 0.373 | 0.071 |
|  | cg15179197 | 13 | 40181094 | *LINC00332* | 0.419 | 0.278 |
|  | cg13729548 | 14 | 92939592 | *ITPK1* | 0.365 | 0.118 |
|  | cg16149919 | 15 | 99005725 | *LOC145814* | 0.491 | 0.139 |
|  | cg08322244 | 17 | 8163351 | *VAMP2* | 0.391 | 0.186 |
|  | cg24298665 | 18 | 50784711 |  | 0.360 | 0.038 |
|  | cg04079758 | 19 | 51006611 | *KLK9* | 0.385 | 0.042 |
|  | cg10451585 | 20 | 4290133 |  | 0.446 | 0.209 |
|  | cg04367531 | 20 | 17885967 |  | 0.345 | 0.104 |
|  | cg15347519 | 20 | 48284092 |  | 0.361 | 0.109 |
|  | | | | | | |

a: Hub CpG sites were highlighted in bold.

b: UCSC GRCh38/hg38

c: Absolute value of module membership in the light-green module. Module membership values above the cutoff were highlighted in bold (|Module membership| ≥ 0.3).

d: Absolute value of gene significance for the cortical thickness of the right postcentral gyrus. Gene significance values above the cutoff were represented as bold (|Gene significance| ≥ 0.9).

CHR, Chromosome

**Table S8. Functional enrichment analysis for genes associated with light-green module (nominal P-value ≤ 0.05, gene count ≥ 2)**

| **Category** | | **ID** | **Description** | **Gene count^a^** | **P-value** |
| --- | --- | --- | --- | --- | --- |
| Gene Ontology | Biological Process (BP) | GO:0016050 | vesicle organization | 3 | 7.25E-03 |
|  |  | GO:0006892 | post-Golgi vesicle-mediated transport | 2 | 8.24E-03 |
|  |  | GO:0006906 | vesicle fusion | 2 | 8.57E-03 |
|  |  | GO:0090174 | organelle membrane fusion | 2 | 8.85E-03 |
|  |  | GO:0048284 | organelle fusion | 2 | 1.35E-02 |
|  |  | GO:0043524 | negative regulation of neuron apoptotic process | 2 | 1.51E-02 |
|  |  | GO:0061025 | membrane fusion | 2 | 1.53E-02 |
|  |  | GO:0050806 | positive regulation of synaptic transmission | 2 | 2.09E-02 |
|  |  | GO:0050804 | modulation of chemical synaptic transmission | 3 | 2.22E-02 |
|  |  | GO:0099177 | regulation of trans-synaptic signaling | 3 | 2.23E-02 |
|  |  | GO:0008361 | regulation of cell size | 2 | 2.68E-02 |
|  |  | GO:1901215 | negative regulation of neuron death | 2 | 2.81E-02 |
|  |  | GO:0043523 | regulation of neuron apoptotic process | 2 | 2.82E-02 |
|  |  | GO:0048167 | regulation of synaptic plasticity | 2 | 3.07E-02 |
|  |  | GO:0090150 | establishment of protein localization to membrane | 2 | 3.17E-02 |
|  |  | GO:0051402 | neuron apoptotic process | 2 | 3.79E-02 |
|  |  | GO:0048193 | Golgi vesicle transport | 2 | 4.61E-02 |
|  |  | GO:0051668 | localization within membrane | 3 | 4.69E-02 |
|  | Cellular Component (CC) | GO:0005802 | trans-Golgi network | 3 | 3.59E-03 |
|  |  | GO:0030667 | secretory granule membrane | 3 | 4.65E-03 |
|  |  | GO:0098791 | Golgi apparatus subcompartment | 3 | 9.32E-03 |
|  |  | GO:0098978 | glutamatergic synapse | 3 | 1.00E-02 |
|  |  | GO:0030141 | secretory granule | 4 | 1.13E-02 |
|  |  | GO:0031984 | organelle subcompartment | 5 | 2.03E-02 |
|  |  | GO:0099503 | secretory vesicle | 4 | 2.35E-02 |
|  |  | GO:0030659 | cytoplasmic vesicle membrane | 4 | 4.81E-02 |
|  | Molecular Function (MF) | GO:0019905 | syntaxin binding | 2 | 3.71E-03 |
|  |  | GO:0000149 | SNARE binding | 2 | 8.41E-03 |
|  |  | GO:0008233 | peptidase activity | 3 | 2.17E-02 |
|  |  | GO:0000287 | magnesium ion binding | 2 | 2.79E-02 |
| a: Number of genes associated with DMPs in each GO term. | | | | | |

**References**

Bibikova, M., Barnes, B., Tsan, C., Ho, V., Klotzle, B., Le, J. M., … Shen, R. (2011). High density DNA methylation array with single CpG site resolution. *Genomics*, *98*(4), 288–295. doi: 10.1016/j.ygeno.2011.07.007

Campagna, M. P., Xavier, A., Lechner-Scott, J., Maltby, V., Scott, R. J., Butzkueven, H., … Lea, R. A. (2021). Epigenome-wide association studies: Current knowledge, strategies and recommendations. *Clinical Epigenetics*, *13*(1), 214. doi: 10.1186/s13148-021-01200-8

Dale, A. M., Fischl, B., & Sereno, M. I. (1999). Cortical surface-based analysis. I. Segmentation and surface reconstruction. *NeuroImage*, *9*(2), 179–194. doi: 10.1006/nimg.1998.0395

Destrieux, C., Fischl, B., Dale, A., & Halgren, E. (2010). Automatic parcellation of human cortical gyri and sulci using standard anatomical nomenclature. *NeuroImage*, *53*(1), 1–15. doi: 10.1016/j.neuroimage.2010.06.010

Fischl, B., Liu, A., & Dale, A. M. (2001). Automated manifold surgery: Constructing geometrically accurate and topologically correct models of the human cerebral cortex. *IEEE Transactions on Medical Imaging*, *20*(1), 70–80. doi: 10.1109/42.906426

Fischl, B., Sereno, M. I., & Dale, A. M. (1999). Cortical surface-based analysis. II: Inflation, flattening, and a surface-based coordinate system. *NeuroImage*, *9*(2), 195–207. doi: 10.1006/nimg.1998.0396

Fischl, Bruce, Salat, D. H., Busa, E., Albert, M., Dieterich, M., Haselgrove, C., … Dale, A. M. (2002). Whole brain segmentation: Automated labeling of neuroanatomical structures in the human brain. *Neuron*, *33*(3), 341–355. doi: 10.1016/s0896-6273(02)00569-x

Fischl, Bruce, van der Kouwe, A., Destrieux, C., Halgren, E., Ségonne, F., Salat, D. H., … Dale, A. M. (2004). Automatically parcellating the human cerebral cortex. *Cerebral Cortex (New York, N.Y.: 1991)*, *14*(1), 11–22. doi: 10.1093/cercor/bhg087

Gonzales, E. L., Jeon, S. J., Han, K.-M., Yang, S. J., Kim, Y., Remonde, C. G., … Shin, C. Y. (2023). Correlation between immune-related genes and depression-like features in an animal model and in humans. *Brain, Behavior, and Immunity*, *113*, 29–43. doi: 10.1016/j.bbi.2023.06.017

Graw, S., Henn, R., Thompson, J. A., & Koestler, D. C. (2019). pwrEWAS: A user-friendly tool for comprehensive power estimation for epigenome wide association studies (EWAS). *BMC Bioinformatics*, *20*(1), 218. doi: 10.1186/s12859-019-2804-7

Han, K.-M., Choi, K. W., Kim, A., Kang, W., Kang, Y., Tae, W.-S., … Ham, B.-J. (2022). Association of DNA Methylation of the NLRP3 Gene with Changes in Cortical Thickness in Major Depressive Disorder. *International Journal of Molecular Sciences*, *23*(10), 5768. doi: 10.3390/ijms23105768

Han, K.-M., Tae, W.-S., Kim, A., Kang, Y., Kang, W., Kang, J., … Ham, B.-J. (2020). Serum FAM19A5 levels: A novel biomarker for neuroinflammation and neurodegeneration in major depressive disorder. *Brain, Behavior, and Immunity*, *87*, 852–859. doi: 10.1016/j.bbi.2020.03.021

Houseman, E. A., Accomando, W. P., Koestler, D. C., Christensen, B. C., Marsit, C. J., Nelson, H. H., … Kelsey, K. T. (2012). DNA methylation arrays as surrogate measures of cell mixture distribution. *BMC Bioinformatics*, *13*(1), 86. doi: 10.1186/1471-2105-13-86

Johnson, W. E., Li, C., & Rabinovic, A. (2007). Adjusting batch effects in microarray expression data using empirical Bayes methods. *Biostatistics*, *8*(1), 118–127. doi: 10.1093/biostatistics/kxj037

Langfelder, P., & Horvath, S. (2008). WGCNA: An R package for weighted correlation network analysis. *BMC Bioinformatics*, *9*(1), 559. doi: 10.1186/1471-2105-9-559

Langfelder, P., Zhang, B., & Horvath, S. (2008). Defining clusters from a hierarchical cluster tree: The Dynamic Tree Cut package for R. *Bioinformatics*, *24*(5), 719–720. doi: 10.1093/bioinformatics/btm563

Mermel, C. H., Schumacher, S. E., Hill, B., Meyerson, M. L., Beroukhim, R., & Getz, G. (2011). GISTIC2.0 facilitates sensitive and confident localization of the targets of focal somatic copy-number alteration in human cancers. *Genome Biology*, *12*(4), R41. doi: 10.1186/gb-2011-12-4-r41

Nordlund, J., Bäcklin, C. L., Wahlberg, P., Busche, S., Berglund, E. C., Eloranta, M.-L., … Syvänen, A.-C. (2013). Genome-wide signatures of differential DNA methylation in pediatric acute lymphoblastic leukemia. *Genome Biology*, *14*(9), r105. doi: 10.1186/gb-2013-14-9-r105

Salas, L. A., Koestler, D. C., Butler, R. A., Hansen, H. M., Wiencke, J. K., Kelsey, K. T., & Christensen, B. C. (2018). An optimized library for reference-based deconvolution of whole-blood biospecimens assayed using the Illumina HumanMethylationEPIC BeadArray. *Genome Biology*, *19*(1), 64. doi: 10.1186/s13059-018-1448-7

Ségonne, F., Pacheco, J., & Fischl, B. (2007). Geometrically accurate topology-correction of cortical surfaces using nonseparating loops. *IEEE Transactions on Medical Imaging*, *26*(4), 518–529. doi: 10.1109/TMI.2006.887364

Teschendorff, A. E., Marabita, F., Lechner, M., Bartlett, T., Tegner, J., Gomez-Cabrero, D., & Beck, S. (2013). A beta-mixture quantile normalization method for correcting probe design bias in Illumina Infinium 450 k DNA methylation data. *Bioinformatics*, *29*(2), 189–196. doi: 10.1093/bioinformatics/bts680

Tian, Y., Morris, T. J., Webster, A. P., Yang, Z., Beck, S., Feber, A., & Teschendorff, A. E. (2017). ChAMP: Updated methylation analysis pipeline for Illumina BeadChips. *Bioinformatics*, *33*(24), 3982–3984. doi: 10.1093/bioinformatics/btx513

Zhou, W., Laird, P. W., & Shen, H. (2017). Comprehensive characterization, annotation and innovative use of Infinium DNA methylation BeadChip probes. *Nucleic Acids Research*, *45*(4), e22. doi: 10.1093/nar/gkw967
